# Multi‐modal adaptor‐clathrin contacts drive coated vesicle assembly

**DOI:** 10.15252/embj.2021108795

**Published:** 2021-09-06

**Authors:** Sarah M Smith, Gabrielle Larocque, Katherine M Wood, Kyle L Morris, Alan M Roseman, Richard B Sessions, Stephen J Royle, Corinne J Smith

**Affiliations:** ^1^ School of Life Sciences University of Warwick Coventry UK; ^2^ Centre for Mechanochemical Cell Biology Warwick Medical School University of Warwick Coventry UK; ^3^ Division of Molecular and Cellular Function School of Biological Sciences Faculty of Biology, Medicine and Health Manchester Academic Health Science Centre University of Manchester Manchester UK; ^4^ School of Biochemistry University of Bristol Bristol UK; ^5^ Present address: Cellular Signalling and Cytoskeletal Function Laboratory The Francis Crick Institute London UK; ^6^ Present address: Diamond Light Source Ltd Harwell Science & Innovation Campus Didcot UK

**Keywords:** clathrin, cryo‐electron microscopy, endocytosis, membrane traffic, Membranes & Trafficking, Structural Biology

## Abstract

Clathrin‐coated pits are formed by the recognition of membrane and cargo by the AP2 complex and the subsequent recruitment of clathrin triskelia. A role for AP2 in coated‐pit assembly beyond initial clathrin recruitment has not been explored. Clathrin binds the β2 subunit of AP2, and several binding sites have been identified, but our structural knowledge of these interactions is incomplete and their functional importance during endocytosis is unclear. Here, we analysed the cryo‐EM structure of clathrin cages assembled in the presence of β2 hinge‐appendage (β2HA). We find that the β2‐appendage binds in at least two positions in the cage, demonstrating that multi‐modal binding is a fundamental property of clathrin‐AP2 interactions. In one position, β2‐appendage cross‐links two adjacent terminal domains from different triskelia. Functional analysis of β2HA‐clathrin interactions reveals that endocytosis requires two clathrin interaction sites: a clathrin‐box motif on the hinge and the “sandwich site” on the appendage. We propose that β2‐appendage binding to more than one triskelion is a key feature of the system and likely explains why assembly is driven by AP2.

## Graphical abstract

Single particle cryo‐EM analysis reveals how Adaptor Protein 2 promotes clathrin assembly during endocytosis by crosslinking clathrin triskelia at different locations in the cage.

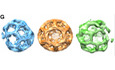

## Introduction

**Figure 1:**
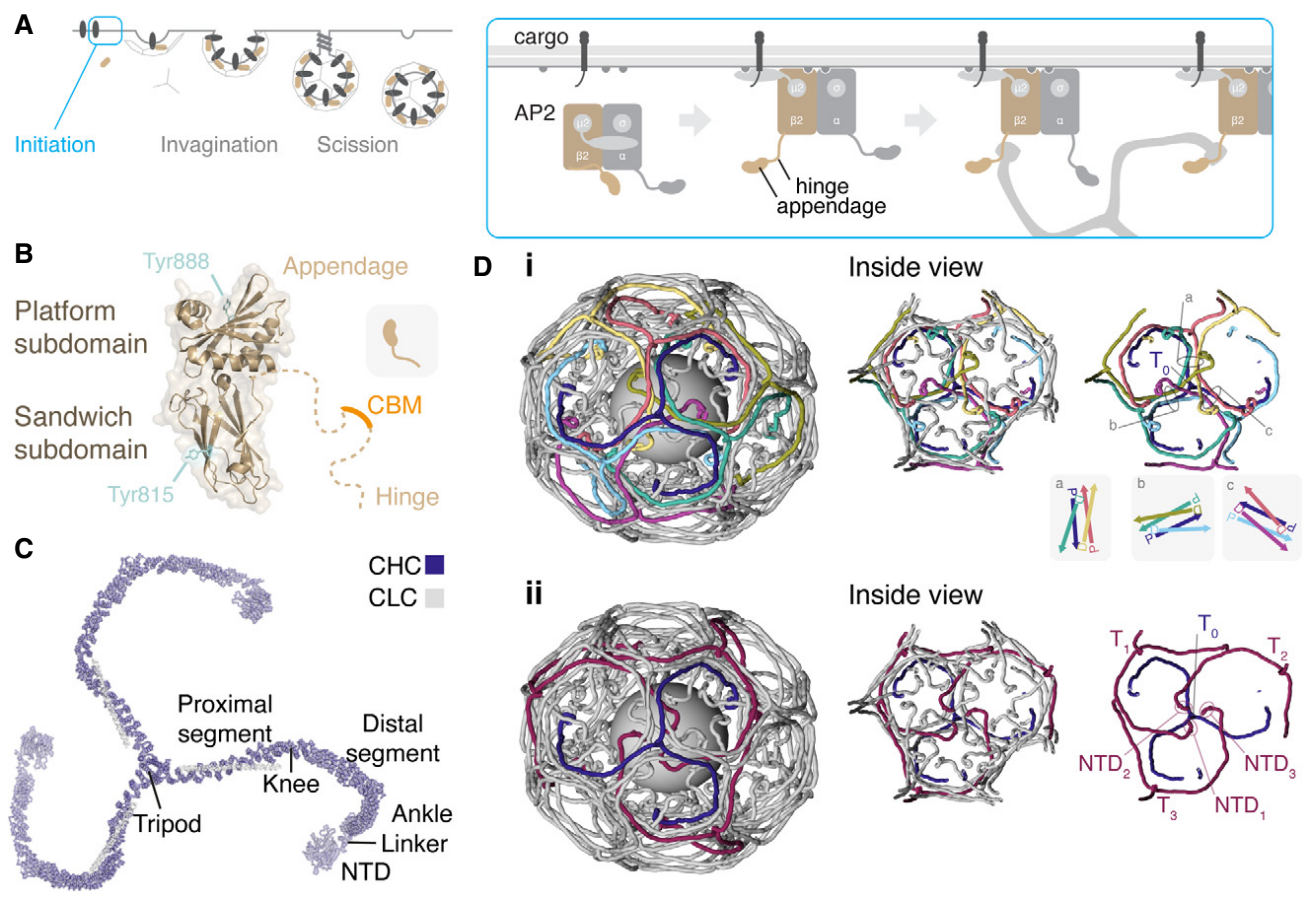
Structural view of clathrin assembly during endocytosis Schematic diagram of clathrin‐mediated endocytosis. The AP2 complex opens when it engages cargo and PI(4,5)P2, the β2 hinge and appendage (β2HA) become available for clathrin binding, initiating pit formation.Structure of β2HA (PDB code: 2G30). The appendage is divided into platform and sandwich subdomain, each with a tyrosine residue previously identified to be important for clathrin binding. The unstructured hinge region contains a clathrin‐box motif (CBM, LLNLD) which binds the N‐terminal domain (NTD) of clathrin heavy chain.Structure of a clathrin triskelion (PDB code: 3IYV). Three clathrin heavy chains (CHC) each with an associated light chain (CLC) are trimerized at their C‐termini forming a tripod. Each leg is divided into proximal and distal segments, an ankle region and NTD.Clathrin assemblies. (i) An indigo triskelion is shown engaged with six other triskelia in a hexagonal barrel, coating a vesicle. Each edge is made from four leg segments for four different triskelia: two antiparallel proximal (P) regions on the outer surface and two antiparallel distal (D) regions below. Three edges (a–c) are shown schematically. (ii) The tripod of this triskelion (T_0_) is at a vertex, and below that, three NTDs (NTD_1‐3_) are arranged, contributed by triskelia (purple) whose tripods are two or three edges away (T_1‐3_). Right panels show the view from the vesicle towards the vertex. The positions of triskelia were mapped by downsampling the carbon backbones in 3IYV by 5 residues and smoothing their position in 3D space using a 25 residue window in IgorPDB. CLCs have been removed for clarity.

Clathrin‐mediated endocytosis (CME) is the major route of entry for receptors and their ligands into cells (Mettlen *et al*, 2018). A clathrin‐coated pit is formed at the plasma membrane that selects cargo for uptake into the cell via a clathrin‐coated vesicle. Clathrin cannot recognize membrane or cargo itself and so an adaptor protein binds the membrane, selects the cargo, and associates with clathrin leading to pit formation (Fig 1A). Several adaptor proteins have clathrin binding sites and colocalize with clathrin structures in cells but the assembly polypeptide‐2 (AP2) complex (α, β2, µ2 and σ2 subunits) is thought to primarily initiate clathrin recruitment.

The recruitment of clathrin by the β2 subunit is an essential step in CME. AP2 and clathrin arrive jointly at the membrane in a ratio of two AP2 complexes per triskelion (Cocucci *et al*, 2012). As the pit matures, the ratio decreases as clathrin polymerizes (Bucher *et al*, 2018). It is assumed that this polymerization—which is an innate property of clathrin triskelia—completes vesicle formation. However, AP2 is named after its ability to promote clathrin cage assembly *in vitro* (Zaremba & Keen, 1983; Pearse & Robinson, 1984), and a fragment of the β2 subunit of AP2, containing the hinge and appendage domains (β2HA), has been shown to promote the polymerization of clathrin (Gallusser & Kirchhausen, 1993; Shih *et al*, 1995; Owen *et al*, 2000). How these *in vitro* observations relate to endocytosis in cells is unclear. One intriguing but often overlooked idea is that AP2, via β2HA, serves a dual role in CME: initially recruiting clathrin to the plasma membrane and then driving coated vesicle assembly.

There are two clathrin‐binding locations on β2HA (Fig 1B). The first is a linear peptide motif within the hinge region (Owen *et al*, 2000; Lundmark & Carlsson, 2002), LLNLD, called the clathrin‐box motif (CBM). The second clathrin‐binding location is within the β2‐appendage domain, however, its precise nature is debated (Chen & Schmid, 2020). The appendage domain has two sites that interact distinctly with different binding partners (Owen *et al*, 2000; Edeling *et al*, 2006; Schmid *et al*, 2006). The first, termed the sandwich (or side) domain, which surrounds Tyr 815, binds AP180, amphiphysin and eps15. A second site, termed the platform (or top) domain, surrounds residues Y888 and W841 (Fig 1B). This binds the adaptor proteins epsin, β‐arrestin and autosomal recessive hypercholesterolemia (ARH) protein and functions independently from the sandwich domain. The roles of these sites in clathrin binding remain to be clarified. *In vitro* pull‐down experiments highlight the potential importance of both Y888 and Y815 for clathrin binding but reports differ on their relative contribution (Owen *et al*, 2000; Edeling *et al*, 2006; Schmid *et al*, 2006).

Our structural understanding of how clathrin engages with AP2 is incomplete. The N‐terminal domain (NTD, Fig 1C) of clathrin heavy chain is a seven‐bladed β‐propeller with four adaptor protein binding sites (Willox & Royle, 2012). Atomic structures have revealed that CBMs bind promiscuously to these sites, with the AP2 CBM binding to the “CBM site” between blades 1 and 2 and also to the “arrestin site” between blades 4 and 5 (Muenzner *et al*, 2017). The location where β2‐appendage binds clathrin is uncertain. Knuehl *et al* (2006) used biochemical approaches and yeast‐2‐hybrid studies to identify residues C682 and G710 on the heavy chain ankle region as a potential location for β2‐appendage. Another potential location is where transforming acidic coiled‐coil 3 (TACC3) binds clathrin (residues 457–507; Burgess *et al*, 2018; Hood *et al*, 2013). However, a full picture of how the β2HA interacts with assembled clathrin, central to the mechanism of clathrin recruitment, remains elusive.

Recently, two structural studies have visualized contradictory modes of binding for the β2‐appendage in clathrin assemblies. Using cryo‐electron tomography, Kovtun *et al* investigated the structure of assembled clathrin and a form of AP2 lacking the alpha appendage and hinge region on lipid membranes containing cargo peptides and PI(4,5)P2 (Kovtun *et al*, 2020). They observed density beneath the clathrin vertex enclosed by one terminal domain and the ankle regions of two triskelion legs (see Fig 1D for orientation). In contrast, Paraan *et al* isolated native coated vesicles from bovine brain and obtained a structure using single particle analysis. They observed density consistent with the β2‐appendage, however it was in a different location, between two adjacent terminal domains (Paraan *et al*, 2020).

In order to address the paradox, we have analysed the structure of purified clathrin bound to the β2HA using single particle cryo‐EM approaches. We find that the β2‐appendage binds in at least two positions on clathrin, within the same sample, demonstrating that multi‐modal binding is a fundamental property of clathrin‐AP2 interactions and reconciling the differing observations in the literature. Our functional analysis of β2HA‐clathrin interactions reveals that endocytosis requires hinge and appendage interaction sites, with the Tyr 815 sandwich site being more important for productive vesicle formation than the Tyr 888 platform site. In consolidating all available structural and functional information, we find that β2‐appendage binding to more than one clathrin triskelion is a key feature of the system and likely explains how clathrin assembly is driven by AP2.

## Results

### The appendage of β2 is critical for coated vesicle formation

**Figure 2:**
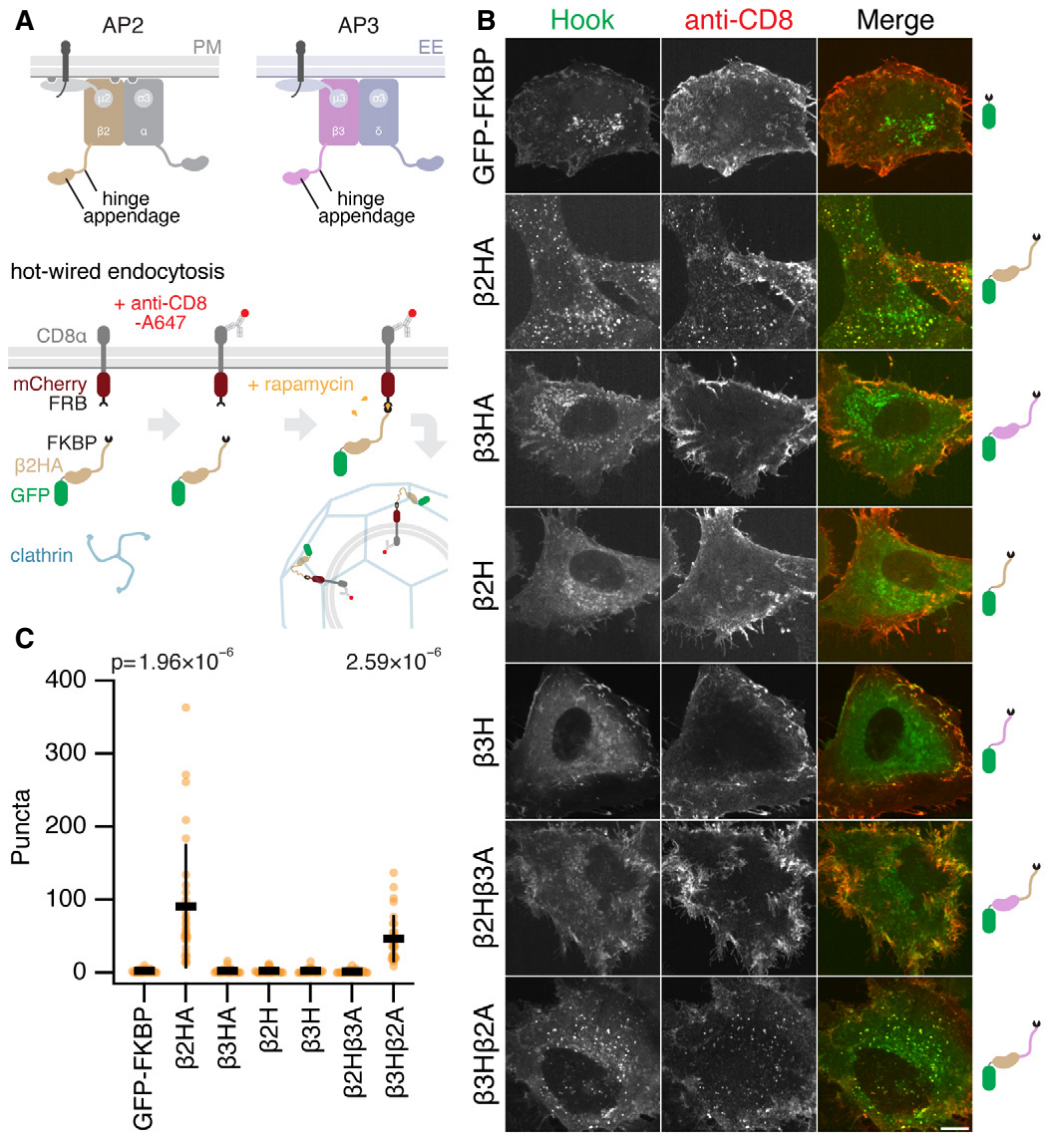
The β2 appendage is crucial for hot‐wired clathrin‐mediated endocytosis Schematic diagram of hot‐wired endocytosis. Under normal conditions the AP2 complex engages with cargo and PI(4,5)P2 at the plasma membrane (PM) and the β2 hinge and appendage become available for clathrin engagement. AP3 acts analogously at the early endosome (EE). In hot‐wired endocytosis a clathrin hook, e.g. β2 hinge and appendage (FKBP‐β2HA‐GFP) is attached to a plasma membrane anchor CD8‐mCherry‐FRB inducibly by rapamycin application. Endocytosis is measured by uptake of a fluorescent antibody that binds the plasma membrane anchor.Representative confocal micrographs of cells expressing the plasma membrane anchor (CD8‐mCherry‐FRB) and the indicated hooks (green). Cells were incubated with anti‐CD8‐Alexa647 (red) and treated with rapamycin (200 nM, 15 min). Endocytic vesicles coinciding in both green and red channels (yellow in merge) were quantified in B. Scale bar, 10 µm.Scatter dot plot shows the number of intracellular GFP‐positive vesicles that contained anti‐CD8 Alexa647 per cell, bars indicate mean ± SD. Number of experiments = 3. *P*‐values from Dunnett’s *post hoc* test that were < 0.1 are shown above.

We previously developed a strategy to trigger clathrin‐coated vesicle formation in cells, termed “hot‐wired endocytosis” (Wood *et al*, 2017). It works by inducibly attaching a clathrin‐binding protein (clathrin “hook”) to a plasma membrane “anchor” using an FKBP‐rapamycin‐FRB dimerization system; and this is sufficient to trigger endocytosis (Fig 2A). Using the hinge and appendage of the β2 subunit of the AP2 complex (FKBP‐β2HA‐GFP) as a clathrin hook allows us to examine endocytosis that is driven by the interaction of β2HA and clathrin, that is, independent of other clathrin‐adaptor interactions. Hot‐wired endocytosis can be detected in live cells by visualizing the formation of intracellular bright green puncta that also contain an antibody to the extracellular portion of the anchor. These puncta move inside the cell, away from the plasma membrane and we have shown previously that they are clathrin‐coated vesicles that have pinched off from the surface and are competent for traffic inside the cell (Wood *et al*, 2017). Using FKBP‐β2HA‐GFP as a clathrin hook, the formation of numerous puncta was observed, while a control construct (FKBP‐GFP) elicited no response (Fig 2B and C).

An analogous construct from the AP3 complex, FKBP‐β3HA‐GFP, with the hinge and appendage of β3, was not competent for hot‐wiring (Fig 2B and C). This is a surprising result for two reasons: first, the clathrin‐box motif in the hinge of β3 binds clathrin *in vitro* (Dell’Angelica *et al*, 1998), and second, we had assumed that the role of the clathrin hook in the hot‐wiring system was solely to recruit clathrin initially, with downstream polymerization being driven by clathrin alone.

To investigate this result in more detail, we tested whether the hinges of β2 or β3 were competent for hot‐wiring. Despite the presence of a clathrin‐box motif in both hinges, with the appendage domains removed neither FKBP‐β2H‐GFP nor FKBP‐β3H‐GFP was able to induce endocytosis (Fig 2B and C). Next, we transplanted the appendage of β3 onto the β2 hinge, and the appendage of β2 onto the β3 hinge. We observed hot‐wiring with FKBP‐β3Hβ2A‐GFP but not with FKBP‐β2Hβ3A‐GFP (Fig 2B and C). Thus, the β2 appendage was able to drive endocytosis with a β3 hinge but the β2 hinge alone or in the presence of the β3 appendage could not. These results indicate firstly that the β2 appendage is critical for endocytosis and that the β3 appendage cannot substitute for this activity. Secondly, hooks containing a clathrin‐box motif are not sufficient for vesicle formation. This suggested to us that the β2 appendage is active in clathrin polymerization.

### Structure of clathrin‐β2HA minicoat cages

**Figure EV1:**
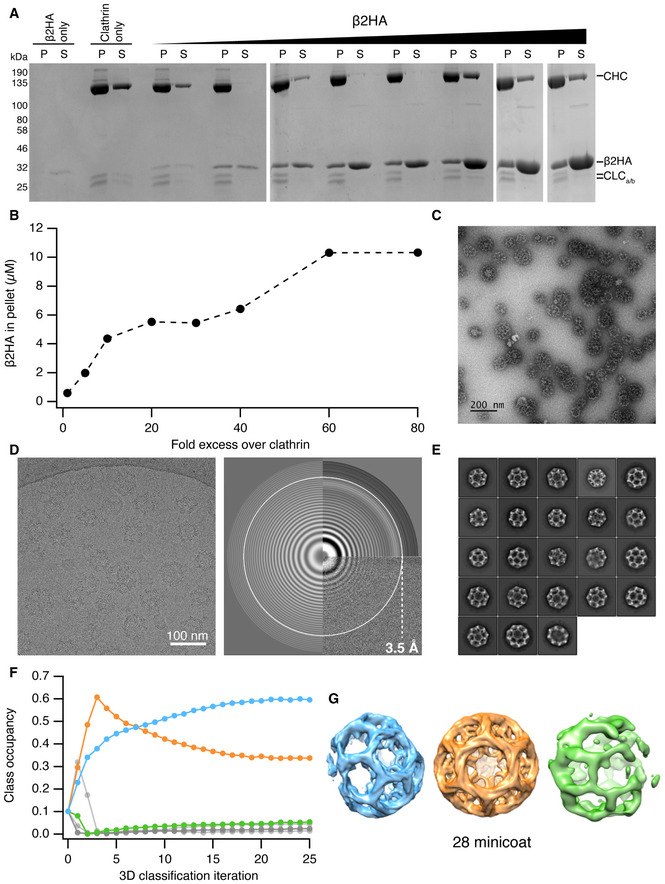
Clathrin‐β2HA reconstitution, data collection and processing Clathrin triskelia (3 µM) were assembled in the presence of increasing concentrations (3–240 µM) of β2‐adaptin_616‐951_ (β2HA). Clathrin assemblies were pelleted and analysed by SDS–PAGE to determine the amount of clathrin (CHC and CLCa/b) and β2HA in the pellet (P) and supernatant (S) fractions.Densitometry of gels in A shows that increasing amounts of β2HA pelleted with clathrin during the reconstitution experiments, with a 60‐fold excess of adaptor protein yielding the maximum amount of clathrin‐binding.Negative stain TEM analysis of clathrin cages reconstituted with a 60‐fold excess (240 µM) of β2HA. Scale bar = 200 nm.A representative cryo‐electron micrograph (left) at −1.4 µm defocus and the corresponding power spectrum indicating the information content at high spatial frequencies (right).2D class averages of classes that were selected for 3D classification in RELION.Particle occupancy of the 10 classes obtained with supervised, asymmetric 3D classification in RELION.3D surface representations of the 3 clathrin cages generated from the supervised, asymmetric 3D classification of clathrin cage particles. Their colour corresponds to the class occupancy data shown in panel F. Only the orange cage was reconstructed in full, enabling its cage geometry to be confirmed as minicoat.

If the β2 appendage contributes to clathrin polymerization, the nature of its interaction with assembled clathrin is of particular interest. In order to investigate this, we analysed cryo‐electron micrographs of clathrin assembled in the presence of β2HA (Fig EV1A–G). Saturation of β2HA binding sites on clathrin was achieved using a 60‐fold molar excess of β2HA (Fig EV1A and B). Of the 57,528 particles analysed, 29% of the total particle data set (16,641 particles) was occupied by the minicoat class of cages (Fig EV1C–G). Subsequent extensive supervised and unsupervised 3D classifications identified the particles most stably associated with the minicoat cage architecture (Appendix Figs S1 and S2). These 13 983 minicoat particles were refined to a gold standard resolution of 9.1 Å (Appendix Fig S3).

**Figure 3:**
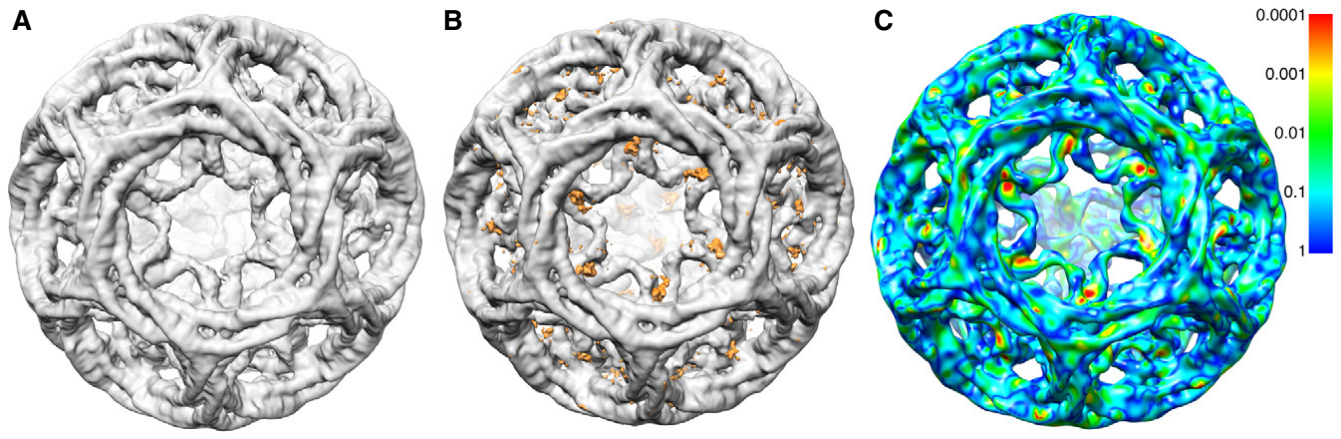
Global difference analysis of clathrin‐β2HA compared to clathrin alone Unsharpened cryo‐EM map of clathrin‐β2HA minicoat cage architecture at 9.1 Å resolution.Difference map of clathrin‐β2HA minicoat and clathrin‐only minicoat. Differences in density are shown in orange.Clathrin‐β2HA and clathrin‐only minicoat maps. Statistically significant differences are shown on a rainbow colour scheme (see inserted panel) with red, orange, yellow and green being the areas of significant difference. The light blue and dark blue areas indicate regions where the significance is below our threshold, or there is no significant difference between the two maps. The regions with the most significant difference density at *P *< 0.0005 (in red) were interpreted as the binding site of β2HA. Other regions show significant differences due to conformation changes related to binding. The contour level of all maps is 3.0 times sigma above the mean of the map. All images were created in UCSF Chimera (Pettersen *et al*, 2004).

In order to locate β2HA within the map density, we compared the β2HA‐clathrin map to a map of clathrin cages assembled in the absence of β2HA. While a difference map did reveal density in a location just above the terminal domains, it was not well‐defined (Fig 3A and B). We therefore conducted a voxel‐by‐voxel comparison between the two maps to locate statistically significant differences (Young *et al*, 2013). This method allows the location of differences to be determined with confidence but does not define the shape of difference density. This enabled us to evaluate the entire minicoat particle data set globally for potential β2HA binding locations. The results of this analysis confirmed a significant difference just above the terminal domains (Fig 3C). We also noted significant differences in some other areas, away from β2HA, that may be related to triskelion leg movements or other conformational changes upon β2HA binding.

### Finding β2HA in clathrin‐β2HA minicoats

**Figure EV2:**
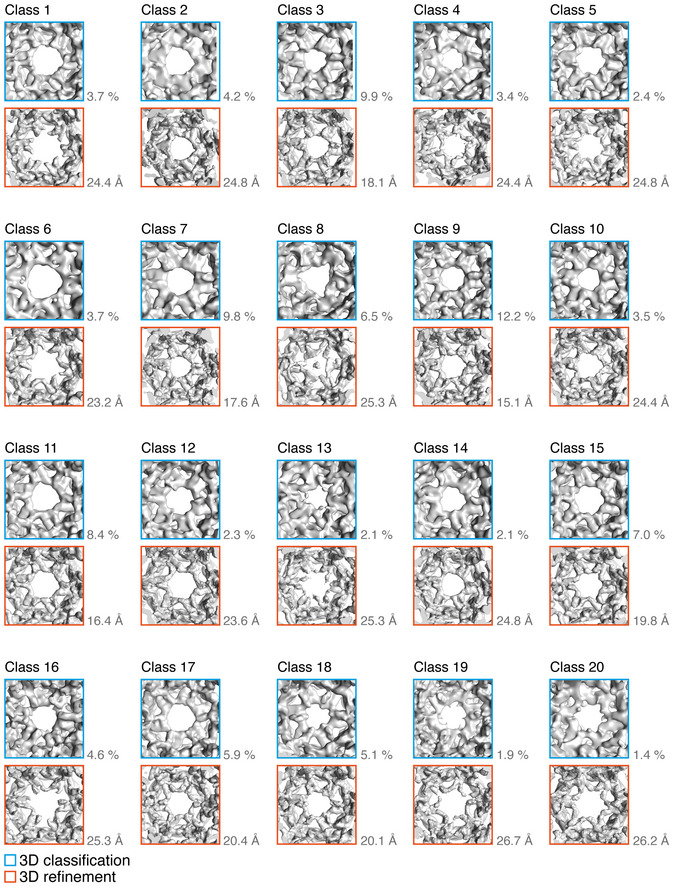
Unsupervised, masked 3D classification of signal‐subtracted minicoat cage particles Output of unsupervised, masked 3D classification of signal‐subtracted minicoat cage particles. Particles were separated into 20 classes: hexagonal faces (representative of average class quality for all remaining polygonal faces) are shown for each class with the corresponding refinement (at 3σ contour level) shown below.

Our global difference analysis suggested that the β2HA was indeed bound to the cages but not well‐resolved. Association of β2HA with clathrin cages may increase sample heterogeneity either through effects on the cage structure itself or through variations in mode of binding, ultimately affecting resolution. In addition, clathrin terminal domain flexibility may result in weaker density in the terminal domain and linker region (Fotin *et al*, 2004; Morris *et al*, 2019). We therefore used signal subtraction to reduce the dominance of the strong features of the outer clathrin cage in order to classify the weaker terminal domain signal more precisely (Bai *et al*, 2015) (Appendix Fig S4). 13,983 particles of the inner region of the minicoat cage were classified into 20 classes, with occupancy ranging from 1.4 to 12.2%, reflecting the heterogeneity of this cage region. Particles belonging to each class were refined individually to a higher resolution (Fig EV2). The outputs of the individual refinements (each at contour level σ3) varied in the quality and completeness of the terminal domain density. However in two classes, 15 and 18, distinct density consistent with bound β2HA was observed.

**Figure EV3:**
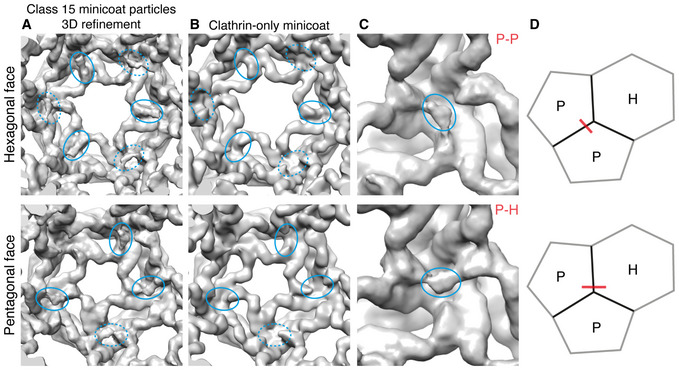
Locating and sub‐classifying β2HA density in class 15 of masked, 3D classification output ARepresentative hexagonal and pentagonal faces for class 15 3D auto refinement. Solid blue ellipses highlight new density seen following masked, 3D classification within a given polygonal face. Dashed ellipses highlight densities connecting adjacent polygonal faces.BRepresentative hexagonal and pentagonal faces for clathrin‐only minicoat cage (low pass filtered to 20 Å). Equivalent positions to those in column A are marked in ellipses, highlighting the lack of density in these regions.C, DDensity cross‐linking terminal domains from two, adjacent pentagonal faces (denoted P‐P) is marked in blue ellipse. Density cross‐linking terminal domains from adjacent hexagonal and pentagonal faces (denoted P‐H) is marked in blue ellipse. The geometric context of P‐P and P‐H densities is depicted in D.

In the case of class 15, these densities were in a different location to that shown by our global analysis, on alternate terminal domains within a polyhedral face (Fig EV3A–D). Comparison of equivalent positions in a minicoat cage without adaptor bound demonstrated that the densities present at the terminal domains were a consequence of β2HA binding (Fig EV3A and B). Looking at adjacent polyhedral faces, for a given hub region where 3 terminal domains (from separate triskelia) converge, two terminal domains are engaged in an interaction with a single β2‐appendage leaving one terminal domain unoccupied (Fig EV3C). Interestingly, β2HA density was not present at any of the 4 hubs in the minicoat cage where 3 pentagonal faces join. This class was refined further using localized reconstruction (described below).

**Figure 4:**
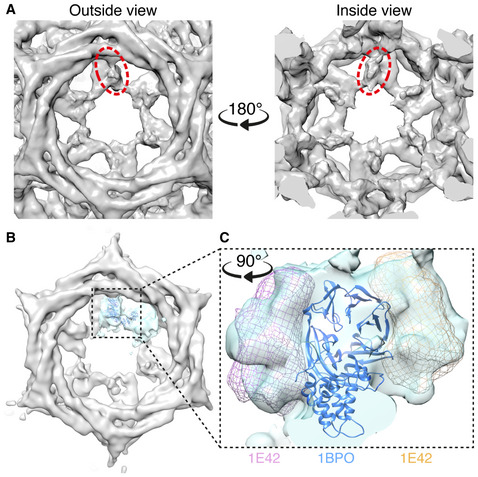
Unattributed density in hexagonal faces of clathrin minicoats Close‐up view of the unattributed density on each terminal domain in a given hexagonal face of the clathrin‐β2HA minicoat cage that was resolved from particles belonging to class 18. Views from outside and inside a given hexagonal face are shown (left and right, respectively). Example densities are highlighted in dashed red ellipses.Using localized reconstruction (Ilca *et al*, 2015), all hexagonal faces from the minicoat cage shown in panel A were extracted and averaged resulting in a 19 Å map. A single terminal domain and connecting linker and ankle region, is highlighted in cyan, with the atomic model of clathrin terminal domain β‐propeller structure (PDB 1BPO) rigid body fitted into the density (shown in dark blue).Rigid‐body fitting of the clathrin terminal domain atomic structure (shown in dark blue) revealed surplus density on either side of the β‐propeller structure, which was large enough to accommodate the atomic structure of the β2‐adaptin appendage (PDB 1E42, shown as pink and orange wireframes) but not sufficiently defined to support an unambiguous fit to the density. All images were created, and rigid‐body fitting was conducted, in UCSF Chimera (Pettersen *et al*, 2004).

For class 18, density could be seen on every terminal domain in all the hexagonal faces of the minicoat volume, but was less well‐resolved (Fig 4A and B). In contrast to class 15 these densities lay parallel to the terminal domain beta‐propeller and did not contact neighbouring terminal domains. We used localized reconstruction (Ilca *et al*, 2015; Morris *et al*, 2019) to improve the resolution of the hexagonal faces from this class of minicoat particles. Rigid‐body fitting of the clathrin terminal domain atomic structure revealed surplus density on either side of the beta‐propeller structure (Fig 4C). The location of this density is consistent with our earlier global difference analysis. The surplus density at either side of the terminal domain was large enough to accommodate the atomic structure of the β2‐appendage (Fig 4C) but could not support an unambiguous fit of this structure.

### Resolving β2HA in the minicoat hub substructure

**Figure EV4:**
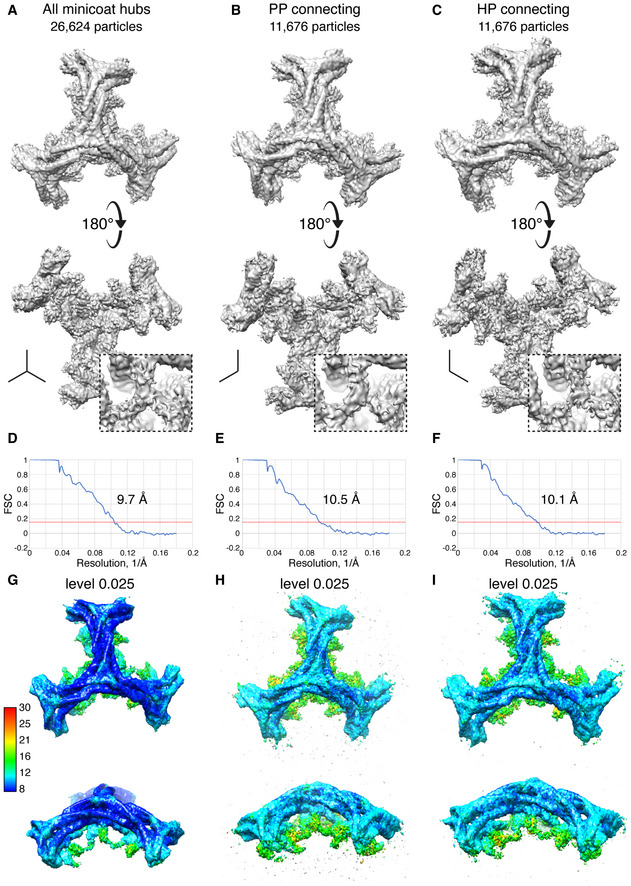
Localized reconstruction of minicoat cage particles AAsymmetric 3D auto refinement of hub regions for all 26,624 minicoat particles from class 15 yielded a 9.7 Å resolution volume. 180° rotation of this asymmetric unit revealed a single β2‐appendage connecting two or three terminal domains (panel below and inset). Local resolution of this region ranged from 12 Å to 16 Å (G).B, CSub‐classification of hub particles based on geometric context. P‐P and H‐P hubs (defined in Fig EV3) yielded volumes resolved at 10.5 Å and 10.1 Å volumes, respectively. The local resolution of the lower hub regions was approximately 16 Å (H and I). Both volumes gave improved definition of the β2‐appendage density.D–FFSC plots for maps shown in A, B and C, respectively. The resolution cut‐off used was at a correlation value of 0.143.G–IShows maps A, B and C, respectively, coloured by local resolution. The scale gives the local resolution in Å.

**Figure 5:**
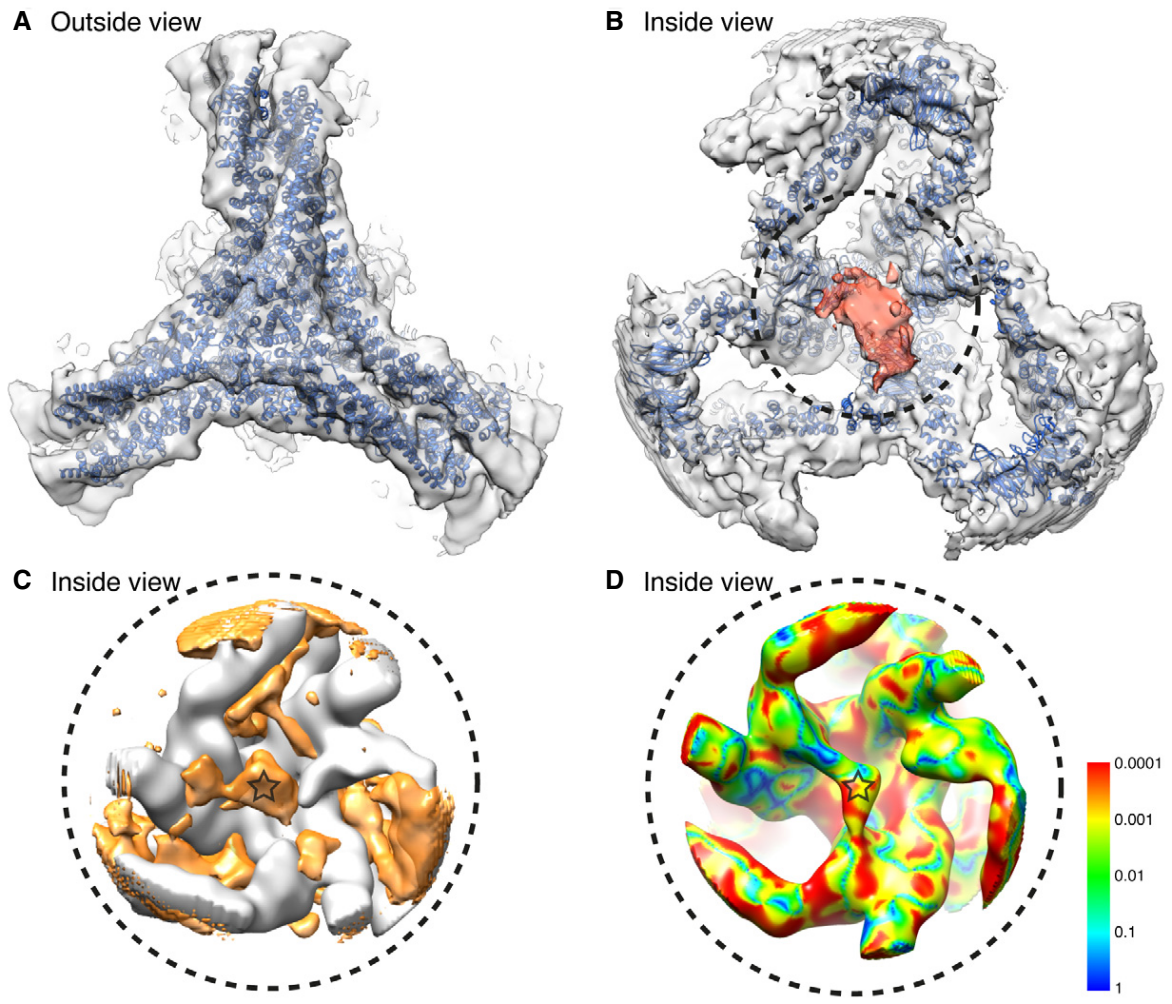
Identification of β2HA in clathrin minicoats 10.5 Å resolution cryo‐EM map of clathrin‐β2HA minicoat hub region particles belonging to class 15. The hub region atomic model (PDB 6SCT) was flexibly fitted (Topf *et al*, 2008; Joseph *et al*, 2016) into the cryo‐EM map (blue).Underside view of the cryo‐EM map and fitted clathrin model shown in panel A. Density attributed to β2HA is coloured red.Difference map of the clathrin‐β2HA minicoat and clathrin‐only minicoat hub maps. Differences in density are shown in orange. The orange density located at the junction of the three terminal domains (marked with a star) is consistent with the location of β2HA shown in panel B.Pixel by pixel comparison between clathrin‐β2HA and clathrin‐only minicoat hub maps. Statistically significant differences are shown on a rainbow colour scheme (see inserted panel) with red, orange, yellow and green being the areas of significant difference. Red indicates the regions with significant differences at the highest level, *P *< 0.0005. Differences reflect the binding of β2HA and induced movements of the legs. The light blue and dark blue areas indicate regions where the significance is below our threshold, or there is no significant difference between the two maps. The density attributed to β2HA is marked with a star.

Having established through our analysis of whole cages that β2HA has at least two different binding locations on assembled clathrin, we next improved the resolution of the most defined density for β2HA by making use of the local symmetry present within the cages. We extracted and refined the hub regions from each vertex of the minicoat cage particles belonging to Class 15 (Fig EV2), using localized reconstruction within RELION (Ilca *et al*, 2015). Using this approach we refined a total of 26,624 minicoat hub regions to a global resolution of 9.6 Å (Fig EV4A–I). This resulted in a considerable improvement in resolution when compared to the whole‐cage particles of Class 15 which refined to 19.8 Å (Fig EV2). Hubs surrounded by three pentagonal faces, which did not show additional density, were excluded from this refinement. A difference map and statistical comparison confirmed the presence of density due to β2HA (Fig 5A–D). We also found that separating the hubs according to whether the β2HA density linked terminal domains emerging from two pentagonal faces or from one pentagonal face and one hexagonal face resulted in improved map definition (Fig EV3C and D). These two classes were refined separately to global resolutions of *∼*10 Å (10.5 Å for P‐P hubs and 10.1 Å for H‐P hubs, Fig EV4B and C), consistent with the reduced number of particles in each subset. Despite this slightly lower resolution, the β2HA density in these maps was more clearly defined than in previous maps, with an intensity equal to the adjoining terminal domains (at contour level σ3), and a 1:2 β2HA:terminal domain binding ratio for both hub volumes (Fig EV4A–C).

### Defining β2‐appendage interactions with clathrin terminal domains

**Figure 6:**
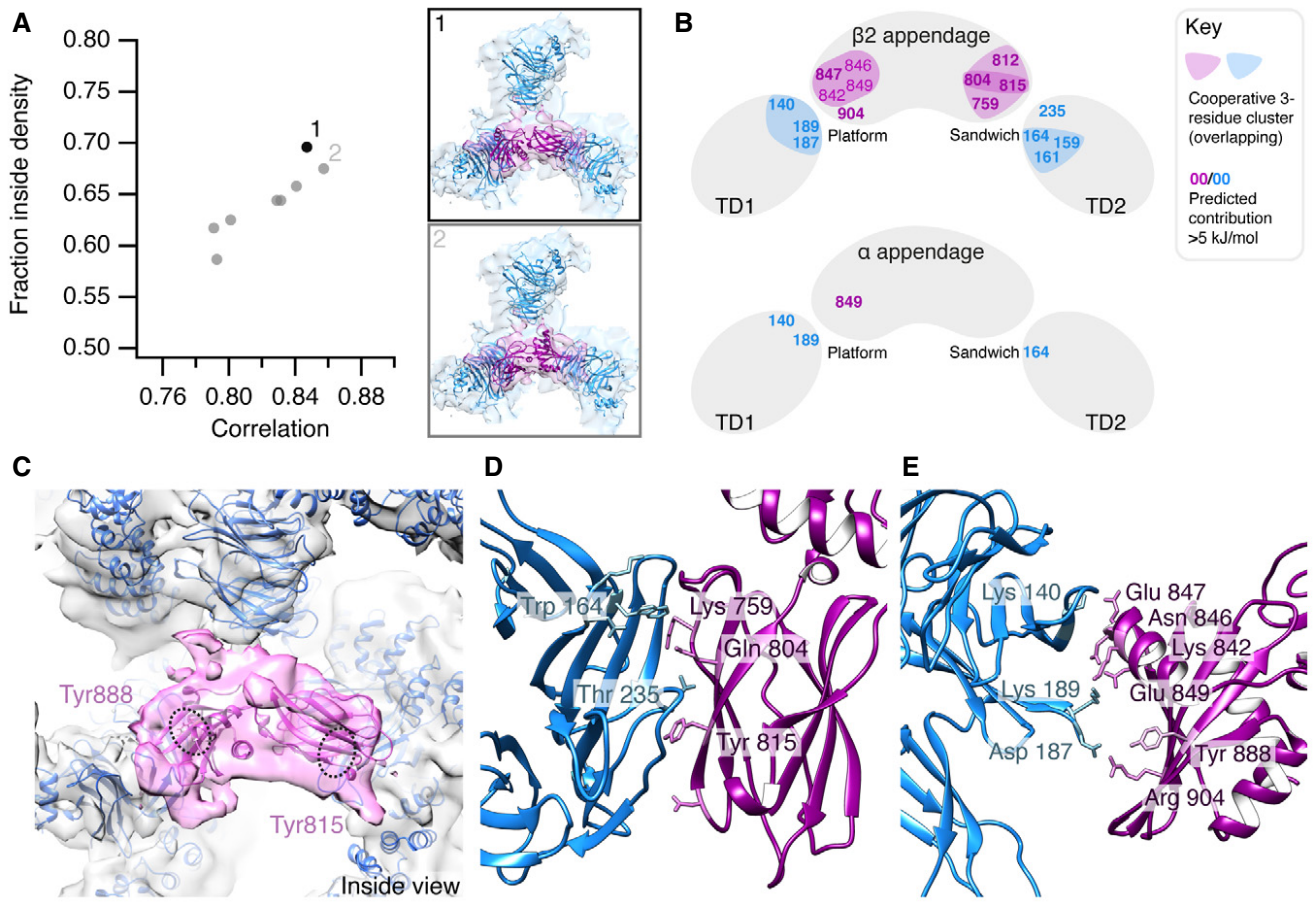
Orientation of β2‐appendage with respect to clathrin terminal domains Plot of fraction of atomic structure inside the cryo‐EM density (Y‐axis) versus correlation of fit (X‐axis). The fit with the largest fraction inside density is shown in black. Although it had the second highest correlation value, this orientation yielded the highest hit‐rate in the rigid body fitting, accounting for 34% of possible fits calculated. Two possible orientations (panels 1 and 2) of the β2‐appendage were determined through rigid body fitting of the atomic structure of Owen *et al*, PDB 1E42 into the P‐P minicoat hub volume from class 15.Diagrammatic summary of the analysis of binding interfaces using BudeAlaScan (BAlaS). Full results are given in Table 1.The selected best fit of the β2‐appendage (purple) is shown in the context of the surrounding clathrin legs (Model in blue, density in grey). The atomic structures of Tyr 815 and Tyr 888 are displayed and highlighted in dashed ellipses. In the orientation shown, Tyr 815 is obscured by the β‐sheets of the β2‐appendage.Predicted interface between terminal domain β‐propeller (blue) and β2‐appendage sandwich or side domain (purple) surrounding Tyr 815.Predicted interface between terminal domain β‐propeller (blue) and β2‐appendage platform or top domain (purple) surrounding Tyr 888.

The improved definition of the β2HA density in the P‐P hubs allowed us to carry out rigid‐body fitting of the atomic structures of β2‐appendage (PDB 1E42; Owen *et al*, 2000) and clathrin terminal domain (PDB 1BPO; ter Haar *et al*, 1998). The optimal orientation of the β2‐appendage was found by selecting the fit with the greatest occupation of density (Fig 6A and C). A molecular model of the clathrin heavy chain formed from the model of Morris *et al* (6SCT) and the terminal domain X‐ray structure of ter Haar *et al* (1BPO) was fitted into the P‐P hub map using a combination of manual fitting and FlexEM (ter Haar *et al*, 1998; Topf *et al*, 2008; Joseph *et al*, 2016; Morris *et al*, 2019). Based on this fit, the alignment of the β2‐appendage and the terminal domains was then optimized according to predicted intermolecular interaction energies calculated using the programme BUDE to determine a plausible binding interface (Fig 6D and E). The resulting model has been deposited as 7OM8.pdb.

We then conducted a systematic analysis of the potential contribution residues at the interface made to binding energy using the programme BudeAlaScan (BAlaS; Ibarra *et al*, 2019; Wood *et al*, 2020) which performs computational alanine scanning (Table 1). In addition to looking at single residues we examined the effect of multiple weaker interactions to define residue clusters that, through a cooperative effect, may prove important for binding. As a control, we performed a similar analysis with the α‐appendage domain which does not bind clathrin. These results predict that residue Tyr 815 on the β2‐appendage makes the largest contribution (14 kJ mol^−1^) with Asp 812, Gln 804 and Lys 759 contributing at or above a 5 kJ mol^−1^ threshold at that interface. There were also contributions from Glu 847 and Arg 904 at the 888 platform domain interface with a second terminal domain. Tyr 888 itself, implicated in adaptor interactions with the β2‐appendage, does not form contacts with the terminal domain in our model. In the control experiments with the α‐appendage, only Glu 849 showed a contribution > 5 kJ mol^−1^ (7.2 kJ mol^−1^).

**Table 1:** Analysis of binding interfaces using BudeAlaScan (BAlaS): alanine scanning.

Protein	Appendage Residue	ΔΔG (kJ mol^−1^)	Appendage Subdomain	TD Residue	ΔΔG (kJ mol^−1^)	Appendage Subdomain, Terminal Domain (TD)
β2 appendage	Tyr 815	14.4	815 sandwich	Thr 235	7.0	815 sandwich, TD chain Y
	Lys 759	6.4	815 sandwich	Trp 164	6.9	815 sandwich, TD chain Y
	Asp 812	5.9	815 sandwich			
	Gln 804	5.1	815 sandwich			
	Glu 847	7.2	888 platform	Lys 140	7.7	888 platform, TD chain Z
	Arg 904	5.8	888 platform	Asp 187	5.7	888 platform, TD chain Z
				Lys 189	5.7	888 platform, TD chain Z
Control. α appendage mapped onto 815 sandwich domain	Glu 849	7.2	888 platform	Trp 164	6.9	815 sandwich, TD chain Y
				Lys 140	7.0	888 platform, TD chain Z
				Lys 189	6.0	888 platform, TD chain Z
Control. α appendage mapped onto 888 platform domain	Glu 849	6.1	888 platform	Lys 189	5.5	888 platform, TD chain Z

A similar analysis looking at the terminal domain interactions showed only two residues contributing more than 5 kJ mol^−1^ to the 815 sandwich interface; Thr 235 and Trp 164, while three terminal domain residues contributed more than 5 kJ mol^−1^ to the 888 platform domain interface; Lys 140, Asp 187 and Lys 189. In the alpha appendage controls, three residues contributed more than 5 kJ mol^−1^; Lys 140, Trp 164 and Lys 189. In all cases, there were no contributions comparable to that of Tyr 815. This suggested that individual residue interactions are less important for terminal domain binding, so we investigated potential cooperativity from groups of weaker‐binding residues. We performed a constellation analysis using BAlaS for residues with an interaction energy greater than 3 kJ mol^−1^. This showed that cooperative clusters formed at the interfaces between the terminal domains and both the 815 sandwich and 888 platform domains (Table 2 and Fig 6B). At the 815 sandwich domain, these complementary clusters involved β2 appendage residues Lys 759, Gln 804, Asp 812 and Tyr 815 and terminal domain residues Asp 159, Lys 161 and Trp 164. At the 888 platform domain interface, the complementary clusters consisted of β2‐appendage residues Lys 842, Asn 846, Glu 847 and Glu 849 and terminal domain residues Lys 140, Asp 187 and Lys 189. For the alpha appendage control, there were no cooperative clusters at the 888 platform domain but at the 815 sandwich domain some pairs of residues showed high cooperativity (Table 2). Interestingly, in our model Lys 140, Lys 189 and Asp 187 form salt bridge contacts with Glu 847, Glu 849 and Arg 904, respectively. In the α‐appendage control, only Glu 849 and Lys 189 form a plausible salt bridge, suggesting a role for Lys 140/Glu 847 and Asp 187/Arg 904 salt bridges in the specificity of this interaction.

**Table 2:** Analysis of binding interfaces using BAlaS: constellation analysis for ∆∆G = 3kJ mol^−1^.

Protein	Constellation	Constellation ΔΔG (kJ mol^−1^)	Summed Individual ΔΔGs (kJ mol^−1^)	Cooperativity (kJ mol^−1^)
β2 appendage (β2‐TD)	B759_B804_B815	9.7	25.8	−16.2
	B804_B812_B815	14.1	25.3	−11.2
	B842_B846_B847	3.8	15.1	−11.3
	B842_B846_B849	−2.4	12.3	−14.7
	B842_B847_B849	1.5	15.2	−13.6
	B846_B847_B849	2.3	16.1	−13.8
TD (β2‐TD)	Y159_Y161_Y164	−1.4	14.7	−16.1
	Z140_Z187_Z189	3.7	19	−15.3
α (α mapped onto 815 sandwich domain)	A761_A763	−15.1	7.7	−22.8
TD (α mapped onto 815 sandwich domain)	Y161_Y164	−13.8	10	−23.8
	Z140_Z189	−8.9	13	−21.9
(α mapped onto 888 platform domain)	n.a.	n.a.	n.a.	n.a.

In all, this analysis of our proposed model suggests that Tyr 815 plays a key role in β2‐appendage clathrin binding, supported by residues Asp 812, Gln 804 and Lys 759. On the terminal domain, residues Thr 235 and Trp 164, supported by Asp 159 and Lys 161 contribute to the interface (Fig 6B,D,E). It also suggests the potential for cooperative clusters of weaker binding interactions to support a binding interface between the 888 platform domain and the terminal domain.

### Role of β2 residues 815 and 888 in functional clathrin assembly

**Figure EV5:**
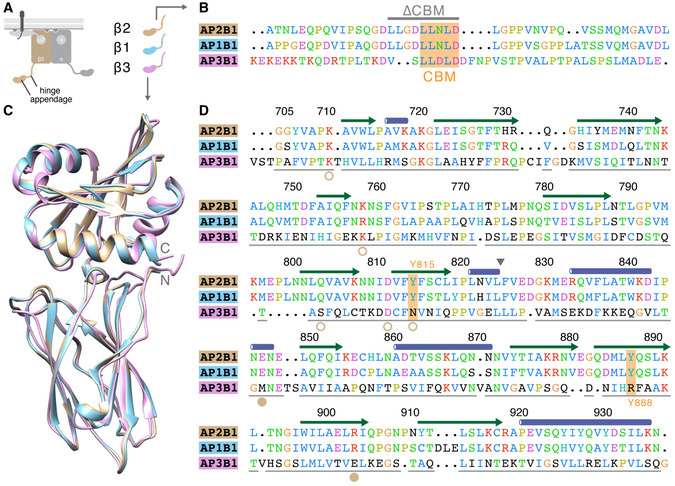
Sequence comparison between β2 and β1 or β3 Schematic diagram of the AP2 complex showing the position of hinge and appendage of the β2 subunit. The colour coding for hinge and appendage of β2, β1 and β3 is shown (left) with the position of the clathrin‐box motif (orange).Alignment of a section of the hinge region of β2, β1 and β3 containing the LL[D/N]LD clathrin‐box motif (orange, CBM). The region deleted in ∆CBM construct is indicated by a grey line. Residues, β2 612‐655, β1 613–658 and β3 835‐883, are coloured according to property. Residues highlighted by BAlaS are indicated (platform residues, filled circles; sandwich residues, open circles).Overlay of β2, β1 and β3 appendage structures. The β2 structure is PDB code 2G30, β1 was created using MODELLER with 2G30 as a template and β3 was created using I‐TASSER using 1E42 as a template.Alignment of the appendage regions shown in C. Structural features and numbering of β2 is shown above. Triangle indicates the separation between the two lobes of the appendage. The position of Tyr 815 and Tyr 888 is indicated in orange. Grey lines indicate structural alignment. Residues are shown coloured by property, and black residues are shown against consensus.

Previous work had identified Tyr 815 and Tyr 888 (shown in Fig 1B) as being important for β2HA‐clathrin interactions (Edeling *et al*, 2006; Schmid *et al*, 2006). Our model and *in silico* alanine scanning analysis had identified the importance of both the platform and sandwich sites on β2‐appendage in this interaction, so we returned to the hot‐wired endocytosis system to address their relative functional importance. We had found that the hinge and appendage of β2 or β1 but not β3, were competent for hot‐wiring (Fig 2B and C; Wood *et al*, 2017). Consistent with these results, Tyr 815 and Tyr 888 are conserved in β2 and β1 but missing in β3 (Fig EV5A–D).

**Figure 7:**
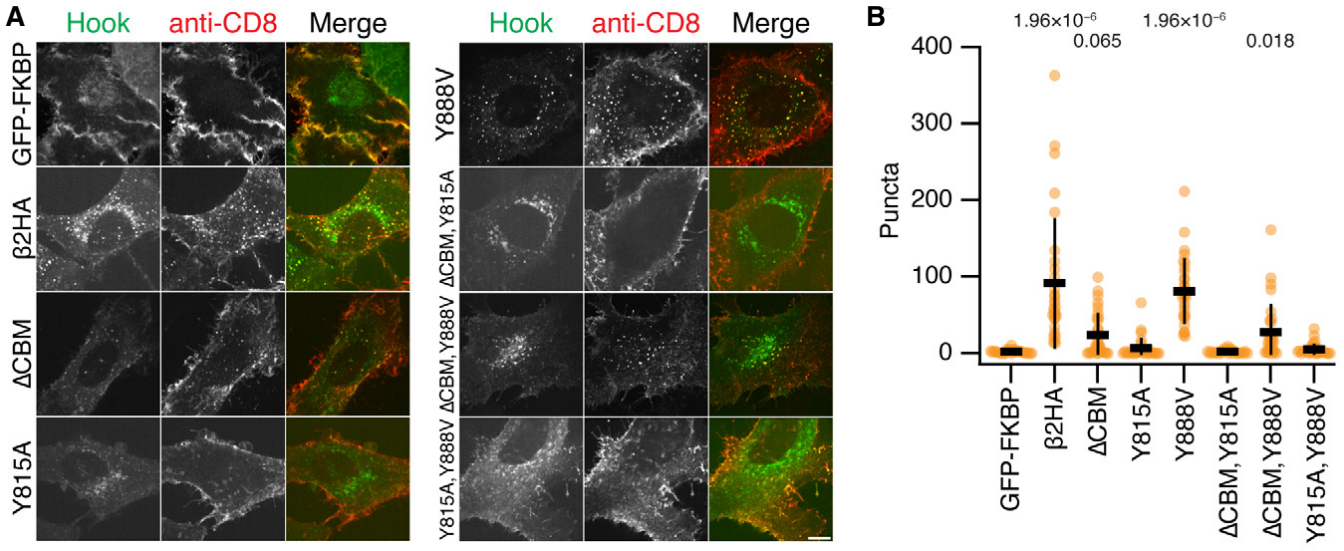
Functional test of importance of Tyr 815 and Tyr 888 to CCV formation Representative confocal micrographs of cells expressing the plasma membrane anchor (CD8‐mCherry‐FRB) and the indicated hooks (green). Cells were incubated with anti‐CD8‐Alexa647 (red) and treated with rapamycin (200 nM, 15 min). Vesicles coinciding in both green and red channels (yellow in merge) were quantified in B. Scale bar, 10 µm.Scatter dot plot shows the number of intracellular GFP‐positive vesicles that contained anti‐CD8 Alexa647 per cell, bars indicate mean ± SD. *P*‐values from Dunnett’s *post hoc* test that were <0.1 are shown above. Note that these results are from the same experimental series as Fig 1 and that the negative and positive control data (GFP‐FKBP and FKBP‐β2HA‐GFP) are as in Fig 1.

Deletion of the clathrin‐box motif (∆CBM) or mutation of Tyr 815 to alanine (Y815A) impaired the ability of FKBP‐β2HA‐GFP to generate endocytic vesicles (Fig 7A and B). Mutation of Tyr 888 to valine (Y888V), a mutation previously reported to reduce clathrin binding (Schmid *et al*, 2006) had no measurable effect on hot‐wiring (Fig 7B). Moreover, the Y888V mutation had no additional effect when combined with ∆CBM, whereas the combination of ∆CBM and Y815A completely ablated hot‐wiring (Fig 7B).

These results suggest that functional clathrin‐β2HA interactions depend on the clathrin‐box motif in the hinge and the sandwich site of the β2 appendage (centred on Tyr 815) while the role of the platform site of the β2 appendage (centred on Tyr 888) is undetectable in this assay.

## Discussion

In this paper, we describe two positions in the clathrin cage where β2‐appendage binds in the assembled state. These occur within the same sample, demonstrating that multi‐modal binding is a fundamental property of clathrin‐AP2 interactions. Our functional analysis demonstrated that endocytosis depends on two interactions between β2HA and clathrin. Together these observations provide an explanation for how AP2 drives coated vesicle formation in cells.

**Figure 8:**
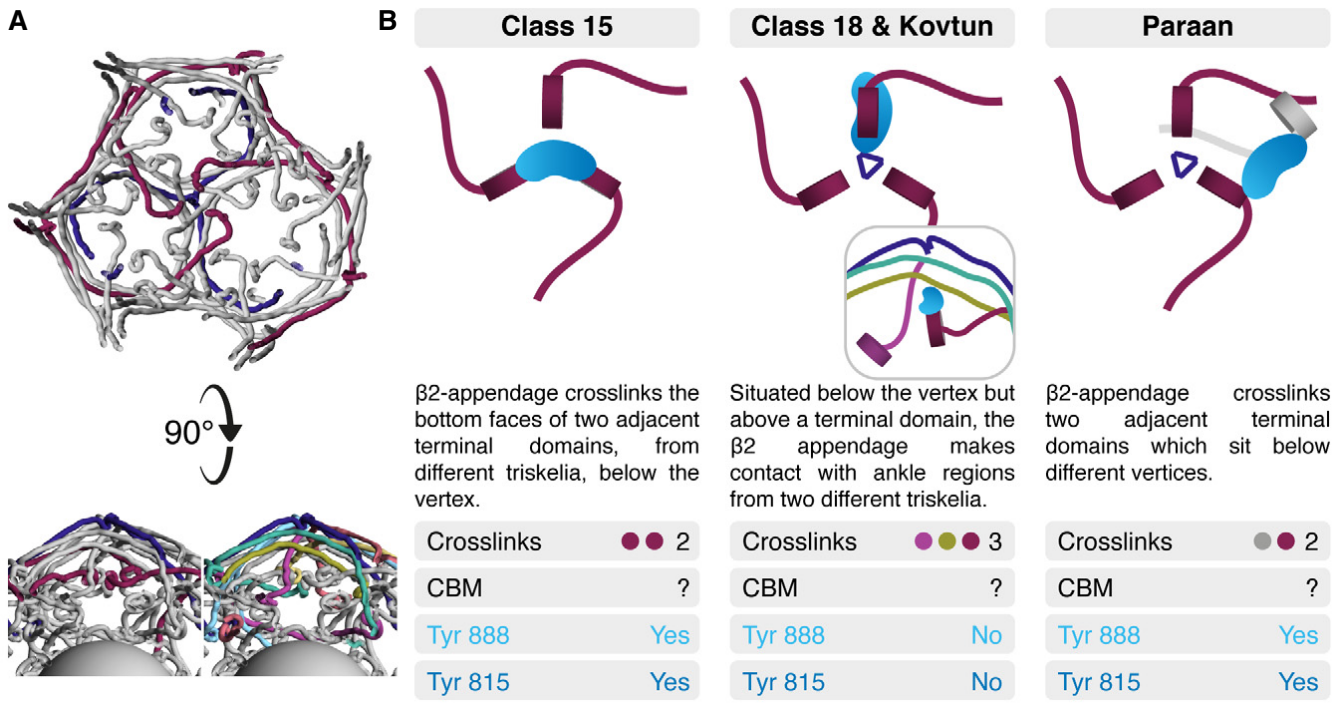
Summary of multi‐modal clathrin‐β2HA interactions For orientation, an indigo triskelion and the three NTDs situated below its vertex are shown, contributed by maroon‐coloured triskelia (viewed from the vesicle, towards the vertex). A “side view” with the same colouring and with alternative colouring (also used in panel B) where the six triskelia that interact with the indigo triskelion are depicted (below).Three modes of binding reported in this study and in two recent studies (Kovtun *et al*, 2020; Paraan *et al*, 2020). Common and contrasting features of each binding mode are shown. The inset in Class 18 & Kovtun shows the side view from A. Each panel indicates the number of cross‐links, whether CBM density was observed and whether Tyr 888 and Tyr815 are in an orientation likely to bind to the terminal domain.

Our observation that β2HA can bind in multiple positions on clathrin within the same sample casts a new perspective on the apparently contradictory observations of Kovtun *et al* (2020) and Paraan *et al* (2020), suggesting they instead form part of a wider spectrum of possible β‐appendage binding modes. We have summarized these multi‐modal clathrin‐β2HA interactions in Fig 8. The first location (Class 15) is between two of the three terminal domains that sit directly beneath the vertex. The second location (Class 18) that we identified is above the terminal domain and maps closely to the location identified by Kovtun *et al*, where the appendage faces CHC repeat 2 from one triskelion and CHC repeat 1 of another. The third location, described by Paraan *et al* is analogous to our Class 15 in that two terminal domains are linked, but they are adjacent domains within a hexagonal face rather than being beneath a cage vertex (Fig 8A and B). A common feature of all three modes is the potential for β2HA to cross‐link clathrin triskelia.

In the first and third modes, the sandwich site and platform site of the β2‐appendage are positioned to make interactions with two distinct terminal domains. Whereas in the second mode, according to the best fit of the β2‐appendage reported by Kovtun *et al* (2020), neither site faces clathrin; although we note that the second highest scoring fit would place the platform site in apposition to CHC repeat 2. This region contains Cys 682 and Gly 710, previously identified as important for binding β2‐ and GGA‐appendages (Knuehl *et al*, 2006).

In comparing our results with those of Kovtun *et al* and Paraan *et al,* we see that differing sample conditions such as inclusion of membrane and cargo (Kovtun *et al*, 2020) or use of coated vesicles purified from source (Paraan *et al*, 2020) do not explain differences in β‐appendage binding location since in our study with only two purified protein components we see multiple binding locations that either confirm (Kovtun *et al*, 2020) or are similar to (Paraan *et al*, 2020) those seen in more complex systems. One interpretation of this is that the long linker domain on β2‐adaptin permits the β‐appendage to move freely, even within a coated vesicle, and form stable interactions unencumbered by the need for additional components or a specific orientation. Further structural and functional studies in systems that more closely reflect the physiological environment will be needed to confirm this.

The location of the β2‐hinge is unknown in all three modes of binding. The clathrin‐box motif in the hinge has been shown to interact as an extended peptide slotted between blades 1 and 2 or between blades 4 and 5 of the beta‐propeller at clathrin heavy chain’s N‐terminal domain (ter Haar *et al*, 2000; Zhuo *et al*, 2015; Muenzner *et al*, 2017). At the current resolution, density relating to a peptide in such an extended conformation would be very hard to distinguish, and the promiscuous nature of clathrin‐box motif binding reduces the observable density further. However, we know that the interaction of this motif is essential for coated vesicle formation possibly because it is involved in initial clathrin recruitment. Assuming that it maintains contact with the terminal domain as the coat forms, all three modes of binding allow for β2HA to make contact with clathrin heavy chains from up to three distinct triskelia.

Our hot‐wiring results indicate that for endocytosis to proceed, the two most important sites on β2HA are the sandwich site and the clathrin‐box motif in the hinge, with no detectable contribution from the platform site. Previous biochemical experiments investigated the importance of Tyr 815 in the sandwich site and Tyr 888 in the platform site in the interaction of β2HA with clathrin (Owen *et al*, 2000; Edeling *et al*, 2006; Schmid *et al*, 2006). Owen *et al* (2000) and Schmid *et al* (2006) showed a significant effect on clathrin binding to the β‐appendage and hinge when Tyr 888 was altered to Val. In experiments where Tyr 815 was altered to Ala, Edeling *et al* (2006) showed that the effect on clathrin binding was most apparent when the hinge region was either absent, or the clathrin‐binding motif within the hinge was deleted or mutated. While these experiments used brain extract for binding, where indirect interactions via other proteins known to bind both clathrin and β2HA remain a possibility, they nonetheless support our conclusion that the sandwich site, in partnership with the CBM domain within the β‐adaptin hinge, is required for clathrin polymerization in functional coat formation. Our *in silico* analysis indicates that, in one mode, the platform site of β2HA does make contact with the terminal domain, and that two pairs of salt bridges (Lys140/Glu847 and Asp187/Arg904) may stabilise this interaction. We interpret our hot‐wiring results to mean that this contact occurs only after binding of the hinge and the sandwich site. Further studies testing the effect of mutagenesis at this interface would help determine whether clathrin binding at this site is of functional importance. Such a cooperative role would allow interaction with other adaptor and accessory proteins such as epsin, β‐arrestin and ARH at the platform domain (Schmid *et al*, 2006), creating flexibility to recruit the additional cargo associated with these adaptors to the growing vesicle.

All available data thus suggest that the β2 subunit of AP2 has a dual function in first recruiting clathrin to the membrane and then driving coated vesicle formation by promoting clathrin polymerization. The alternative model, where β2 only recruits clathrin and clathrin self‐assembles in the absence of contribution of the adaptors, is highly unlikely. First, a single clathrin‐box motif, which is sufficient to bind clathrin *in vitro* is not sufficient to induce coated vesicle formation in the hot‐wiring assay. Second, under the alternative model, the requirement for the appendage in addition to the hinge would mean that both interactions must occur on one triskelion exclusively. The multiple modes of binding described from cryo‐EM data all feature triskelia cross‐linking and therefore make this model improbable.

The multi‐modal nature of β2HA‐clathrin interactions that cross‐link triskelia raise the question of whether other adaptors have the same property. Alternative adaptors such as epsin, β‐arrestin and AP180 have multiple motifs that interact with clathrin or with AP2 (Traub, 2009; Smith *et al*, 2017). This suggests that they may also be able to contribute to initial recruitment and to cross‐linking during assembly. In the case of epsin, the linear unstructured domain alone is capable of forming coated pits *in vitro* (Dannhauser & Ungewickell, 2012) and forming vesicles in cells using hot‐wired endocytosis (Wood *et al*, 2017). The interpretation of these experiments was simply that epsin recruited clathrin and then clathrin self‐assembled into a cage. It is possible that this region of epsin may also cross‐link triskelia and thereby contribute to coat formation. If this is the case, it suggests a mechanism whereby adaptor proteins included in a growing coat can enhance clathrin polymerization and thereby stabilize, or even accelerate, coated vesicle formation. Since epsin, β‐arrestin and AP180 also bring cargo to the growing vesicle, this has implications for understanding how particular cargos may, through their associated adaptor, increase the likelihood of completion of a growing vesicle and consequently drive forward their own internalization.

## Materials and Methods

### Molecular biology

The hinge and appendage of the human β2 subunit of the AP2 complex (designated β2HA), corresponding to residues 616–951 of the long isoform, was used for all experiments. Numbering of residues in the appendage is after the structure of β2‐appendage which uses the numbering of the shorter isoform, ending at residue 937 (Owen *et al*, 2000).

CD8‐mCherry‐FRB, FKBP‐β2HA‐GFP, FKBP‐β3HA‐GFP, FKBP‐β2HA(Y815A)‐GFP, FKBP‐β2HA(∆CBM)‐GFP and FKBP‐β2HA(∆CBM,Y815A)‐GFP were described previously (Wood *et al*, 2017). The Y888V mutation was added by site‐directed mutagenesis to FKBP‐β2HA‐GFP, FKBP‐β2HA(Y815A)‐GFP and FKBP‐β2HA(∆CBM)‐GFP. FKBP‐β2H‐GFP was made by substituting β2‐hinge (616–704) in place of β2HA in FKBP‐β2HA‐GFP using BamHI and AgeI. Similarly, FKBP‐β3H‐GFP was made by substituting β3‐hinge (702–859) in FKBP‐β3HA‐GFP using PspOMI and AgeI. FKBP‐β2Hβ3A‐GFP was made by inserting the β3‐appendage (860–1,094) into FKBP‐β2A‐GFP via SalI and NotI. FKBP‐β3Hβ2A‐GFP was made by inserting the β2‐appendage (705–951) into FKBP‐β3 hinge‐GFP via SalI and NotI. Plasmid to express GST‐β2HA in bacteria was available from previous work (Hood *et al*, 2013).

### Cell culture and light microscopy

HeLa cells (Health Protection Agency/European Collection of Authenticated Cell Cultures, #93021013) were kept in DMEM supplemented with 10% FBS and 100 U ml^−1^ penicillin/streptomycin at 37°C and 5% CO_2_. DNA transfection was performed with Genejuice (Merck Millipore) using the manufacturer’s protocol. HeLa cells were transfected with CD8‐mCherry‐FRB and one of the hooks (GFP‐FKBP, FKBP‐β2HA‐GFP, FKBP‐β3HA‐GFP, FKBP‐β2H‐GFP, FKBP‐β3H‐GFP, FKBP‐β2Hβ3A‐GFP, FKBP‐β3Hβ2A‐GFP, FKBP‐β2HA(∆CBM)‐GFP, FKBP‐β2 HA(Y815A)‐GFP, FKBP‐β2HA(Y888V)‐GFP, FKBP‐β2HA(∆CBM,Y815A)‐GFP, FKBP‐β2HA(∆CBM,Y888V)‐GFP or FKBP‐β2HA(Y815A,Y888V)‐GFP).

After 24 h, the cells were put on coverslips. The next day, surface CD8 was labelled with 10 µg ml^−1^ AlexaFluor647‐anti‐CD8 antibody (Bio‐Rad, MCA1226A647) at 37°C for 5 min. To induce dimerization of the hook to the CD8, the medium was changed for DMEM with 200 nm rapamycin (Alfa Aesar) for 15 min at 37°C. The cells were then fixed with fixation buffer (4% formaldehyde, 4% sucrose, 80 mM K‐PIPES, 5 mM EGTA, 2 mM MgCl_2_, pH 6.8) for 10 min at RT. The coverslips were rinsed 4 × 5 min with PBS and mounted in Mowiol and DAPI.

Cells were imaged using a spinning disc confocal system (Ultraview Vox; PerkinElmer) with a 100× 1.4 NA oil‐immersion objective. Images were captured in Volocity using a dual‐camera system (ORCA‐R2; Hamamatsu) after excitation with lasers of wavelength 488 and 640 nm.

### Image analysis

The images acquired were duplicated and thresholded to isolate vesicular structures. To analyse only coinciding structures, thresholded images were multiplied with one another using the “Image calculator” plugin in FIJI and the vesicular structures measuring between 0.03 µm^2^ and 0.8 µm^2^ and of 0.3‐1 circularity were counted in the resulting image using the “analyse particles” plugin. A one‐way ANOVA with Dunnett’s *post hoc* test was performed using GFP‐FKBP as control.

### Buffer compositions

HKM buffer: 25 mM HEPES pH 7.2, 125 mM potassium acetate, 5 mM magnesium acetate. Tris buffer: 1 M Tris pH 7.1, 1 mM EDTA, 1 mM DTT. Ficoll/Sucrose buffer: 6.3% w/v Ficoll PM 70, 6.3% w/v sucrose in HKM pH 7.2. Saturated ammonium sulphate: excess ammonium sulphate dissolved in 10 mM Tris pH 7, 0.1 mM EDTA. Polymerization buffer: 100 mM MES pH 6.4, 1.5 mM MgCl_2_, 0.2 mM EGTA. Depolymerization buffer: 20 mM TEA pH 8.0, 1 mM EDTA, 1 mM DTT. Purification buffer: 20 mM HEPES pH 7.2, 200 mM NaCl. Elution buffer: 20 mM HEPES pH 7.0, 200 mM NaCl, 10 mM reduced glutathione. Prescission buffer: 50 mM tris–HCl pH 7.0, 150 mM NaCl, 1 mM EDTA, 1 mM DTT.

### Protein expression and purification

Clathrin was purified from pig brain clathrin‐coated vesicles (see detailed method in Rothnie *et al*, 2011 Supporting Information). Clathrin cages were assembled for harvesting by dialyzing the purified triskelia into polymerization buffer at 4°C and then harvested by ultracentrifugation. Pellets containing clathrin cages were resuspended in a small volume of polymerization buffer. Concentration of clathrin cages was assayed by A_280_ of triskelia to avoid the effects from light scattering.

β2HA was expressed as a GST‐β2HA fusion protein in *Escherichia coli* strain, BL21. Bacteria were grown at 37°C to an OD_600_ of 0.6 and then induced with 0.8 mM IPTG at 20°C overnight. Cells were harvested and resuspended in purification buffer (supplemented with Complete Protease Inhibitor Cocktail tablet as per Roche Applied Science instructions) and lysed by sonication. The soluble fraction was obtained by centrifugation at 75,400 *g* for 30 min. GST‐β2HA was purified from the soluble fraction using glutathione resin (GE Healthcare) and GST‐β2HA was displaced from the glutathione resin using elution buffer. The GST tag was removed by a GST fusion 3C protease (Prescission, GE Healthcare) by dialysing GST‐β2HA with GST fusion 3C protease, in prescission buffer, overnight at 4°C. The fusion protease was removed using glutathione resin and the cleaved β2HA was collected in the flow‐through. Cleaved β2HA was concentrated and loaded onto a HiLoad Superdex 200 (equilibrated in purification buffer) for further purification via size exclusion chromatography. Fractions containing purified β2HA were pooled and concentrated on Vivaspin columns (Sartorius).

### β2HA‐clathrin complex preparation

To identify the maximum amount of β2HA that could bind the clathrin cages, increasing molar amounts of β2HA was reconstituted with 3 µM clathrin in depolymerization buffer at 4°C and subsequent dialysis overnight into polymerization buffer at 4°C. The β2HA‐clathrin cage complexes were harvested by centrifugation at 230,000 *g* for 30 min and concentrated 10‐fold by pellet resuspension into a small volume of polymerization buffer. The protein composition of the resuspended pellets was analysed by SDS–PAGE and densitometry in ImageJ (Schneider *et al*, 2012).

### Negative stain transmission electron microscopy

Clathrin cages reconstituted in the presence of 180 µM β2HA were screened under negative stain. Assembled β2HA‐clathrin cage complexes (5 µl of 1 µM) were pipetted onto a glow‐discharged formvar carbon 300‐mesh copper grid (Agar Scientific) and incubated for 1 min at room temperature. Excess sample was removed by blotting with Whatman filter paper and 5 µl of 1% (w/v) uranyl acetate stain was subsequently applied to the grid and left to incubate for 1 min at room temperature. Excess negative stain was removed by blotting with Whatman filter paper. Samples were imaged using a JEOL 2100Plus and Gatan OneView IS at 200 keV.

### Cryo‐electron microscopy

3 µl of β2HA clathrin cage complexes (clathrin at 9 µM) were applied to glow‐discharged 300‐mesh copper Quantifoil R1.2/1.3 grids and blotted at 4°C and > 90% humidity for 4.5 s before plunging into an ethane/propane mix (80%/20%) liquefied and cooled by liquid nitrogen using a Leica EM GP automated plunge freezing device.

Cryo‐electron micrographs were collected as movies using a Titan Krios and Falcon III detector (Leicester Institute of Structural and Chemical Biology), operating at 300 keV. EPU was used for automated data collection, movies were acquired at a total dose of 64 e^–^ Å^−2^ over 1 s at a dose rate of 1.65 e^–^ Å^−2^ s^−1^ with a magnified pixel size of 1.39 Å px^−1^ using a 1 µm beam and 70 µm C2 aperture. Three images were acquired per hole with some illumination of the carbon support. Micrographs were targeted for collection between 1.1 and 2 µm defocus.

### Data processing

Beam‐induced motion of the specimen was anisotropically corrected, with and without dose‐weighting, using MotionCor2 (Li *et al*, 2013). The contrast transfer function of the motion‐corrected summed micrographs was estimated from non‐dose‐weighted micrographs using gctf v1.06 (Zhang, 2016) employing equiphase averaging and validation functions. RELION v3.0.5 (Scheres, 2012) was used for particle picking, extraction, and all classifications and refinements. 57,528 particles were manually picked from the non‐dose‐weighted, motion‐corrected micrographs and then extracted at a binned pixel size of 10.8 Å px^−1^. Reference‐free 2D classification, over 25 iterations, was first used to analyse the quality of the extracted particles. The highest quality classes, containing 51,133 particles, were selected for further 3D classification. As previously described, supervised asymmetric 3D classification successfully sorted the particles into ten structural classes (Morris *et al*, 2019). The particles associated with the minicoat cage type, which produced the highest quality 3D classification output, were selected for subsequent hierarchical, supervised 3D classifications to identify the particles most stably associated with this particular cage geometry (Appendix Fig S1). Further unsupervised 3D classification of these stable minicoat particles subdivided the particles into 3 classes and was carried out using a regularization parameter (*T*) of 4, no imposed symmetry and no mask (Appendix Fig S2). 3D auto refinement of the most stable minicoat particles (at 10.8 Å px^−1^, without symmetry imposed) yielded a 24 Å minicoat volume. These particles were reextracted from non‐dose‐weighted micrographs with a box size of 500 px and a pixel size of 2.78 Å px^−1^; large enough to include clathrin cages over 1,000 Å diameter. The minicoat cage architecture was refined at 2.78 Å px^−1^ (i.e. binned twofold) without imposing symmetry. The refinement reference (from the previous 3D auto refinement of minicoat particles) was low pass filtered to 40 Å. Since the output volume was a minicoat with mixed handedness, an unsupervised 3D classification was conducted on the minicoat particles (no symmetry imposed, and no alignment of particles). Only the minicoat particles contributing to volumes that had 100% surface density were saved and used in subsequent processing. These particles were refined as described for the previous 3D auto refinement, and yielded a 11.7 Å volume. A mask was generated from this C_1_ reconstruction at 3σ, extended and softened by 2 and 9 px. This mask was employed in subsequent C_1_ refinements that used dose‐weighted minicoat particles, solvent flattening and a Gaussian noise background. Reconstructions with and without imposed symmetry correlated well.

Resolution of each reconstruction was estimated using the gold standard Fourier shell correlation (FSC) measurement within a mask created from the refinement volume (using threshold value of 3σ, expanded by 2 px to 4 px and softened by 9 px). The MTF of the Falcon III camera (operated at 300 keV) was applied and the B factor of the map was automatically calculated if the resolution exceeded 10 Å. In instances, where sub‐10 Å refinements were calculated, a user‐defined B factor value was given.

In order to identify β2HA in the minicoat volume we subtracted the signal contributed by the outer coat region and subsequently conducted a masked, unsupervised 3D classification on the signal‐subtracted inner cage region (Appendix Fig S4) with a regularization parameter (*T*) of 20 tetrahedral (T) symmetry imposed and no alignment of particles calculated. The mask was created from the tetrahedral refinement volume using a threshold value of 3σ, expanded by 5 px and softened by 9 px. The particles contributing to each of the 20 classes were saved separately and refined with *T* symmetry imposed. Qualitative analysis of the individual refinement outputs (visualized at contour level 3σ), identified two classes that possessed strong additional density that was not present in reconstructions calculated using signal from the whole cage (i.e. prior to signal subtraction) *or* in a minicoat cage volume reconstructed *without* adaptor protein present.

### Localized subparticle extraction and reconstruction

To improve the resolution of the strong additional density resolved after masked 3D classifications of signal‐subtracted minicoat particles, we performed localized reconstruction (Ilca *et al*, 2015) as previously described for single particle data sets of clathrin cages (Morris *et al*, 2019). Hub regions were extracted and recentred as new subparticles in 350 px boxes from whole minicoat cage particles. Each of the extracted hub subparticles were reconstructed separately to serve as references in subsequent refinements.

All refinements were conducted in C_1_ with masking applied from a 3σ extended 2 px and softened 9 px mask (3σ/e2/s9). Global resolution of the hub region was estimated as described previously using the gold standard FSC approach (within a mask 3σ/e2/s9). The refinement was found to have converged at 9.6 Å. Local resolution estimations were made using ResMap (Kucukelbir *et al*, 2014) revealing lower resolutions in the terminal domain regions of the minicoat hub. To improve the quality of the β2HA density located between the terminal domains under the hub vertex, the hub subparticles were classified based on whether the β2HA density connected terminal domains from two separate pentagonal faces (PP) or connected a hexagonal and pentagonal face (HP). Compared to the whole‐cage volume (post‐signal subtraction), the resolution of the β2HA (and neighbouring clathrin heavy chain regions) is improved, allowing PDBs of the clathrin heavy chain (residues 1–361, 362–487 and 488–834) to be fitted into the hub volume.

### Global difference analysis

Student’s *t*‐test was used to determine the significance of differences between two structures, using SPIDER and the programmes of Milligan and Flicker as previously published (Milligan & Flicker, 1987; Frank *et al*, 1996; Young *et al*, 2013). In order to do this, independent maps of each structure (four in the case of whole cages, three for the hubs) were created using RELION. The ‐split command in the relion_star_handler script was used to divide the data into separate sets, taking care to distribute images of particles evenly from each micrograph and therefore the defocus spread. A low pass Fourier filter, 11 Å in the case of whole cages and 12 Å for the hubs, was applied to the maps. In order to avoid potential false differences due to variations in the quality of the two structures solved, or random effects such as the sampling of defocus values, the two structures were scaled together in reciprocal space by calculating their radial amplitude‐profiles. A reciprocal‐space scaling profile was calculated by comparing the amplitude profile of the clathrin‐only map with the β‐adaptin map (Young *et al*, 2013). Using this, all the β‐adaptin sub‐maps were rescaled to fit the profile of the clathrin‐only map. These maps were used to calculate an average and variance for each structure. The per voxel value of *t* and the significance of differences was computed from these, using the appropriate degrees of freedom. Many regions had significant differences with *P *< 0.05. Regions we have interpreted to show direct density differences relating to ligand binding have *P *< 0.0001. The images show the original maps, with the value of *P* coloured onto the surface according to the scale shown.

### Redocking the adaptor proteins

Initially, the β2‐appendage protein structure (1E42) was docked by fitting into the unoccupied density in the β2HA‐clathrin map. However, this led to an overlap of residues between the C‐terminal domain and its neighbouring terminal domain, while leaving a gap at the putative interface between the N‐terminal domain and a second local terminal domain. The docking programme BUDE (McIntosh‐Smith *et al*, 2012, 2015) was used to refine this structure as follows. The complex was centred on the centre‐of‐coordinates of the β2‐appendage and the complex split into clathrin as the receptor and β2‐appendage as the ligand. The docking grid was defined: −10 to 10 in 2° increments for rotation and −10 to 10 in 1 Å increments for translation. A genetic algorithm, EMC (Abraham *et al*, 2015), sampling 1.1 million poses was used to find low energy poses.

The best pose was inspected and new rotamers chosen for a few interfacial sidechains to optimize putative interfacial interactions (β2‐appendage: R732, R759, N846, E849, E882; clathrin: R188) and the above docking procedure repeated. Next, Gromacs 2019.4 (Abraham *et al*, 2015) was used to parameterize the complex with the Amber99SB‐ildn (Lindorff‐Larsen *et al*, 2010) forcefield at pH 7 and place it in box of TIP3P water containing 0.15 m NaCl. A short energy minimization (200 steps of steepest descents) was performed to remove bad intermolecular atom‐atom contacts, permitted by BUDE’s very soft empirical free energy forcefield, and yield the finished model.

The initial α‐appendage complex was prepared by superimposing the α‐appendage (1B9K) on the β2‐appendage C‐terminal domain of the finished complex. This preliminary model of the α‐appendage complex was subjected to the same docking and minimization procedure described above. Because the angle between the platform and sandwich domains of the α‐appendage is smaller, we also prepared complexes where either the platform or sandwich domain of the α‐appendage (1B9K) were directly superimposed onto the corresponding region of the β2‐appendage domain of the finished complex using the Matchmaker function in UCSF Chimera.

### *In silico* alanine scanning

The two energy‐minimized complexes and the individual platform and sandwich domain α‐appendage complexes were presented to the BAlaS server http://balas.app (Wood *et al*, 2020). The three clathrin domains were assigned as the receptor and the appendages (either β2 or α) as the ligand. Alanine scanning and constellation calculations were performed and the results downloaded.

### Figure preparation

Maps and models were visualized using UCSF Chimera (Pettersen *et al*, 2004). Simplified views of triskelia were generated using IgorPDB and 3IYV. Microscopy figures and plots were made Fiji and Igor Pro (WaveMetrics Inc.). All figures were assembled in Adobe Illustrator.

## Author contributions

SMS carried out structural biology experiments and contributed to the manuscript writing and figure preparation. GL conducted hot‐wiring experiments contributed to the manuscript writing and figure preparation. KMW carried out structural biology experiments and contributed to the manuscript writing. KLM contributed to structural analysis and manuscript writing. AMR carried out structure comparisons and contributed to the manuscript writing. RBS carried out model building and contributed to the manuscript writing. SJR contributed to data interpretation, manuscript writing and figure preparation. CJS contributed to data interpretation, structural analysis and wrote the final draft, which was approved by all authors.

## Conflict of interest

The authors declare that they have no conflict of interest.

## Supporting information



Appendix

Expanded View Figures PDF

## Data Availability

EM maps supporting this study have been deposited in the Electron Microscopy Data Bank with accession codes EMD‐12980, EMD‐12981, EMD‐12983 and EMD‐12984 (relating to Figs 4B and C, 4A, 3 and 5, respectively). Particle stacks associated with EMD‐12980, EMD‐12983 and EMD‐12984 were deposited to EMPIAR as 10784, 10779 and 10783, respectively. The fitted model of clathrin terminal domains and β2‐appendage has been deposited in the protein data bank as 7OM8.pdb. Original models of both the clathrin terminal domain [1BPO.pdb, (ter Haar *et al*, 1998)] and β2‐appendage [1E42.pdb, (Owen *et al*, 2000)] were used to generate the fitted model with only the interfaces between the protein molecules remodelled. No inter‐molecule clashes have been identified in the fitted model. The intra‐molecule clashes and geometric outliers are historical.

## References

[embj2021108795-bib-0001] Abraham MJ, Murtola T, Schulz R, Páll S, Smith JC, Hess B, Lindahl E (2015) GROMACS: high performance molecular simulations through multi‐level parallelism from laptops to supercomputers. SoftwareX 1–2: 19–25

[embj2021108795-bib-0002] Bai X, Rajendra E, Yang G, Shi Y, Scheres SHW (2015) Sampling the conformational space of the catalytic subunit of human γ‐secretase. Elife 4: e11182 26623517 10.7554/eLife.11182 PMC4718806

[embj2021108795-bib-0003] Bucher D, Frey F, Sochacki KA, Kummer S, Bergeest J‐P, Godinez WJ, Kräusslich H‐G, Rohr K, Taraska JW, Schwarz US*et al* (2018) Clathrin‐adaptor ratio and membrane tension regulate the flat‐to‐curved transition of the clathrin coat during endocytosis. Nat Commun 9: 1109 29549258 10.1038/s41467-018-03533-0 PMC5856840

[embj2021108795-bib-0004] Burgess SG, Mukherjee M, Sabir S, Joseph N, Gutiérrez‐Caballero C, Richards MW, Huguenin‐Dezot N, Chin JW, Kennedy EJ, Pfuhl M*et al* (2018) Mitotic spindle association of TACC3 requires Aurora‐A‐dependent stabilization of a cryptic α‐helix. EMBO J 37: e97902 29510984 10.15252/embj.201797902 PMC5897774

[embj2021108795-bib-0005] Chen Z, Schmid SL (2020) Evolving models for assembling and shaping clathrin‐coated pits. J Cell Biol 219: e202005126 32770195 10.1083/jcb.202005126 PMC7480099

[embj2021108795-bib-0006] Cocucci E, Aguet F, Boulant S, Kirchhausen T (2012) The first five seconds in the life of a clathrin‐coated pit. Cell 150: 495–507 22863004 10.1016/j.cell.2012.05.047 PMC3413093

[embj2021108795-bib-0007] Dannhauser PN, Ungewickell EJ (2012) Reconstitution of clathrin‐coated bud and vesicle formation with minimal components. Nat Cell Biol 14: 634–639 22522172 10.1038/ncb2478

[embj2021108795-bib-0008] Dell’Angelica EC, Klumperman J, Stoorvogel W, Bonifacino JS (1998) Association of the AP‐3 adaptor complex with clathrin. Science 280: 431–434 9545220 10.1126/science.280.5362.431

[embj2021108795-bib-0009] Edeling MA, Mishra SK, Keyel PA, Steinhauser AL, Collins BM, Roth R, Heuser JE, Owen DJ, Traub LM (2006) Molecular switches involving the AP‐2 beta2 appendage regulate endocytic cargo selection and clathrin coat assembly. Dev Cell 10: 329–342 16516836 10.1016/j.devcel.2006.01.016

[embj2021108795-bib-0010] Fotin A, Cheng Y, Sliz P, Grigorieff N, Harrison SC, Kirchhausen T, Walz T (2004) Molecular model for a complete clathrin lattice from electron cryomicroscopy. Nature 432: 573–579 15502812 10.1038/nature03079

[embj2021108795-bib-0011] Frank J, Radermacher M, Penczek P, Zhu J, Li Y, Ladjadj M, Leith A (1996) SPIDER and WEB: processing and visualization of images in 3D electron microscopy and related fields. J Struct Biol 116: 190–199 8742743 10.1006/jsbi.1996.0030

[embj2021108795-bib-0012] Gallusser A, Kirchhausen T (1993) The beta 1 and beta 2 subunits of the AP complexes are the clathrin coat assembly components. EMBO J 12: 5237–5244 8262066 10.1002/j.1460-2075.1993.tb06219.x PMC413789

[embj2021108795-bib-0013] ter Haar E, Harrison SC, Kirchhausen T (2000) Peptide‐in‐groove interactions link target proteins to the beta‐propeller of clathrin. Proc Natl Acad Sci USA 97: 1096–1100 10655490 10.1073/pnas.97.3.1096 PMC15533

[embj2021108795-bib-0014] ter Haar E, Musacchio A, Harrison SC, Kirchhausen T (1998) Atomic structure of clathrin. Cell 95: 563–573 9827808 10.1016/s0092-8674(00)81623-2 PMC4428171

[embj2021108795-bib-0015] Hood FE, Williams SJ, Burgess SG, Richards MW, Roth D, Straube A, Pfuhl M, Bayliss R, Royle SJ (2013) Coordination of adjacent domains mediates TACC3‐ch‐TOG‐clathrin assembly and mitotic spindle binding. J Cell Biol 202: 463–478 23918938 10.1083/jcb.201211127 PMC3734082

[embj2021108795-bib-0016] Ibarra AA, Bartlett GJ, Hegedüs Z, Dutt S, Hobor F, Horner KA, Hetherington K, Spence K, Nelson A, Edwards TA*et al* (2019) Predicting and experimentally validating hot‐spot residues at protein‐protein interfaces. ACS Chem Biol 14: 2252–2263 31525028 10.1021/acschembio.9b00560 PMC6804253

[embj2021108795-bib-0017] Ilca SL, Kotecha A, Sun X, Poranen MM, Stuart DI, Huiskonen JT (2015) Localized reconstruction of subunits from electron cryomicroscopy images of macromolecular complexes. Nat Commun 6: 8843 26534841 10.1038/ncomms9843 PMC4667630

[embj2021108795-bib-0018] Joseph AP, Malhotra S, Burnley T, Wood C, Clare DK, Winn M, Topf M (2016) Refinement of atomic models in high resolution EM reconstructions using Flex‐EM and local assessment. Methods 100: 42–49 26988127 10.1016/j.ymeth.2016.03.007 PMC4854230

[embj2021108795-bib-0019] Knuehl C, Chen C‐Y, Manalo V, Hwang PK, Ota N, Brodsky FM (2006) Novel binding sites on clathrin and adaptors regulate distinct aspects of coat assembly. Traffic 7: 1688–1700 17052248 10.1111/j.1600-0854.2006.00499.x

[embj2021108795-bib-0020] Kovtun O, Dickson VK, Kelly BT, Owen DJ, Briggs JAG (2020) Architecture of the AP2/clathrin coat on the membranes of clathrin‐coated vesicles. Sci Adv 6: eaba8381 32743075 10.1126/sciadv.aba8381 PMC7375805

[embj2021108795-bib-0021] Kucukelbir A, Sigworth FJ, Tagare HD (2014) Quantifying the local resolution of cryo‐EM density maps. Nat Methods 11: 63–65 24213166 10.1038/nmeth.2727 PMC3903095

[embj2021108795-bib-0022] Li X, Mooney P, Zheng S, Booth CR, Braunfeld MB, Gubbens S, Agard DA, Cheng Y (2013) Electron counting and beam‐induced motion correction enable near‐atomic‐resolution single‐particle cryo‐EM. Nat Methods 10: 584–590 23644547 10.1038/nmeth.2472 PMC3684049

[embj2021108795-bib-0023] Lindorff‐Larsen K, Piana S, Palmo K, Maragakis P, Klepeis JL, Dror RO, Shaw DE (2010) Improved side‐chain torsion potentials for the Amber ff99SB protein force field. Proteins 78: 1950–1958 20408171 10.1002/prot.22711 PMC2970904

[embj2021108795-bib-0024] Lundmark R, Carlsson SR (2002) The beta‐appendages of the four adaptor‐protein (AP) complexes: structure and binding properties, and identification of sorting nexin 9 as an accessory protein to AP‐2. Biochem J 362: 597–607 11879186 10.1042/0264-6021:3620597 PMC1222423

[embj2021108795-bib-0025] McIntosh‐Smith S, Price J, Sessions RB, Ibarra AA (2015) High performance in silico virtual drug screening on many‐core processors. Int J High Perform Comput Appl 29: 119–134 25972727 10.1177/1094342014528252 PMC4425459

[embj2021108795-bib-0026] McIntosh‐Smith S, Wilson T, Ibarra AA, Crisp J, Sessions RB (2012) Benchmarking energy efficiency, power costs and carbon emissions on heterogeneous systems. Comput J 55: 192–205

[embj2021108795-bib-0027] Mettlen M, Chen P‐H, Srinivasan S, Danuser G, Schmid SL (2018) Regulation of clathrin‐mediated endocytosis. Annu Rev Biochem 87: 871–896 29661000 10.1146/annurev-biochem-062917-012644 PMC6383209

[embj2021108795-bib-0028] Milligan RA, Flicker PF (1987) Structural relationships of actin, myosin, and tropomyosin revealed by cryo‐electron microscopy. J Cell Biol 105: 29–39 3611188 10.1083/jcb.105.1.29 PMC2114877

[embj2021108795-bib-0029] Morris KL, Jones JR, Halebian M, Wu S, Baker M, Armache J‐P, Avila Ibarra A, Sessions RB, Cameron AD, Cheng Y*et al* (2019) Cryo‐EM of multiple cage architectures reveals a universal mode of clathrin self‐assembly. Nat Struct Mol Biol 26: 890–898 31582853 10.1038/s41594-019-0292-0 PMC7100586

[embj2021108795-bib-0030] Muenzner J, Traub LM, Kelly BT, Graham SC (2017) Cellular and viral peptides bind multiple sites on the N‐terminal domain of clathrin. Traffic 18: 44–57 27813245 10.1111/tra.12457 PMC5182127

[embj2021108795-bib-0031] Owen DJ, Vallis Y, Pearse BM, McMahon HT, Evans PR (2000) The structure and function of the beta 2‐adaptin appendage domain. EMBO J 19: 4216–4227 10944104 10.1093/emboj/19.16.4216 PMC302036

[embj2021108795-bib-0032] Paraan M, Mendez J, Sharum S, Kurtin D, He H, Stagg SM (2020) The structures of natively assembled clathrin‐coated vesicles. Sci Adv 6: eaba8397 32743076 10.1126/sciadv.aba8397 PMC7375819

[embj2021108795-bib-0033] Pearse BM, Robinson MS (1984) Purification and properties of 100‐kd proteins from coated vesicles and their reconstitution with clathrin. EMBO J 3: 1951–1957 6149117 10.1002/j.1460-2075.1984.tb02075.x PMC557627

[embj2021108795-bib-0034] Pettersen EF, Goddard TD, Huang CC, Couch GS, Greenblatt DM, Meng EC, Ferrin TE (2004) UCSF Chimera–a visualization system for exploratory research and analysis. J Comput Chem 25: 1605–1612 15264254 10.1002/jcc.20084

[embj2021108795-bib-0035] Rothnie A, Clarke AR, Kuzmic P, Cameron A, Smith CJ (2011) A sequential mechanism for clathrin cage disassembly by 70‐kDa heat‐shock cognate protein (Hsc70) and auxilin. Proc Natl Acad Sci USA 108: 6927–6932 21482805 10.1073/pnas.1018845108 PMC3084117

[embj2021108795-bib-0036] Scheres SHW (2012) RELION: implementation of a Bayesian approach to cryo‐EM structure determination. J Struct Biol 180: 519–530 23000701 10.1016/j.jsb.2012.09.006 PMC3690530

[embj2021108795-bib-0037] Schmid EM, Ford MGJ, Burtey A, Praefcke GJK, Peak‐Chew S‐Y, Mills IG, Benmerah A, McMahon HT (2006) Role of the AP2 beta‐appendage hub in recruiting partners for clathrin‐coated vesicle assembly. PLoS Biol 4: e262 16903783 10.1371/journal.pbio.0040262 PMC1540706

[embj2021108795-bib-0038] Schneider CA, Rasband WS, Eliceiri KW (2012) NIH Image to ImageJ: 25 years of image analysis. Nat Methods 9: 671–675 22930834 10.1038/nmeth.2089 PMC5554542

[embj2021108795-bib-0039] Shih W, Gallusser A, Kirchhausen T (1995) A clathrin‐binding site in the hinge of the beta 2 chain of mammalian AP‐2 complexes. J Biol Chem 270: 31083–31090 8537368 10.1074/jbc.270.52.31083

[embj2021108795-bib-0040] Smith SM, Baker M, Halebian M, Smith CJ (2017) Weak molecular interactions in clathrin‐mediated endocytosis. Front Mol Biosci 4: 72 29184887 10.3389/fmolb.2017.00072 PMC5694535

[embj2021108795-bib-0041] Topf M, Lasker K, Webb B, Wolfson H, Chiu W, Sali A (2008) Protein structure fitting and refinement guided by cryo‐EM density. Structure 16: 295–307 18275820 10.1016/j.str.2007.11.016 PMC2409374

[embj2021108795-bib-0042] Traub LM (2009) Tickets to ride: selecting cargo for clathrin‐regulated internalization. Nat Rev Mol Cell Biol 10: 583–596 19696796 10.1038/nrm2751

[embj2021108795-bib-0043] Willox AK, Royle SJ (2012) Functional analysis of interaction sites on the N‐terminal domain of clathrin heavy chain. Traffic 13: 70–81 21939487 10.1111/j.1600-0854.2011.01289.x PMC3365446

[embj2021108795-bib-0044] Wood CW, Ibarra AA, Bartlett GJ, Wilson AJ, Woolfson DN, Sessions RB (2020) BAlaS: fast, interactive and accessible computational alanine‐scanning using BudeAlaScan. Bioinformatics 36: 2917–2919 31930404 10.1093/bioinformatics/btaa026

[embj2021108795-bib-0045] Wood LA, Larocque G, Clarke NI, Sarkar S, Royle SJ (2017) New tools for ‘hot‐wiring’ clathrin‐mediated endocytosis with temporal and spatial precision. J Cell Biol 216: 4351–4365 28954824 10.1083/jcb.201702188 PMC5716275

[embj2021108795-bib-0046] Young A, Stoilova‐McPhie S, Rothnie A, Vallis Y, Harvey‐Smith P, Ranson N, Kent H, Brodsky FM, Pearse BMF, Roseman A*et al* (2013) Hsc70‐induced changes in clathrin‐auxilin cage structure suggest a role for clathrin light chains in cage disassembly. Traffic 14: 987–996 23710728 10.1111/tra.12085 PMC3776051

[embj2021108795-bib-0047] Zaremba S, Keen JH (1983) Assembly polypeptides from coated vesicles mediate reassembly of unique clathrin coats. J Cell Biol 97: 1339–1347 6138359 10.1083/jcb.97.5.1339 PMC2112702

[embj2021108795-bib-0048] Zhang K (2016) Gctf: Real‐time CTF determination and correction. J Struct Biol 193: 1–12 26592709 10.1016/j.jsb.2015.11.003 PMC4711343

[embj2021108795-bib-0049] Zhuo Y, Cano KE, Wang L, Ilangovan U, Hinck AP, Sousa R, Lafer EM (2015) Nuclear magnetic resonance structural mapping reveals promiscuous interactions between clathrin‐box motif sequences and the N‐terminal domain of the clathrin heavy chain. Biochemistry 54: 2571–2580 25844500 10.1021/acs.biochem.5b00065 PMC4429812

