# Supplementary material for: Multi‐modal adaptor‐clathrin contacts drive coated vesicle assembly
Source: EMBO J. 2021 Sep 6;40(19):e108795. doi: 10.15252/embj.2021108795 (PMC8488560; doi:10.15252/embj.2021108795)
Supplement: Supplementary file 1 — Appendix [file EMBJ-40-e108795-s002.pdf]

Appendix

Appendix Figure S1
Appendix Figure S2
Appendix Figure S3
Appendix Figure S4

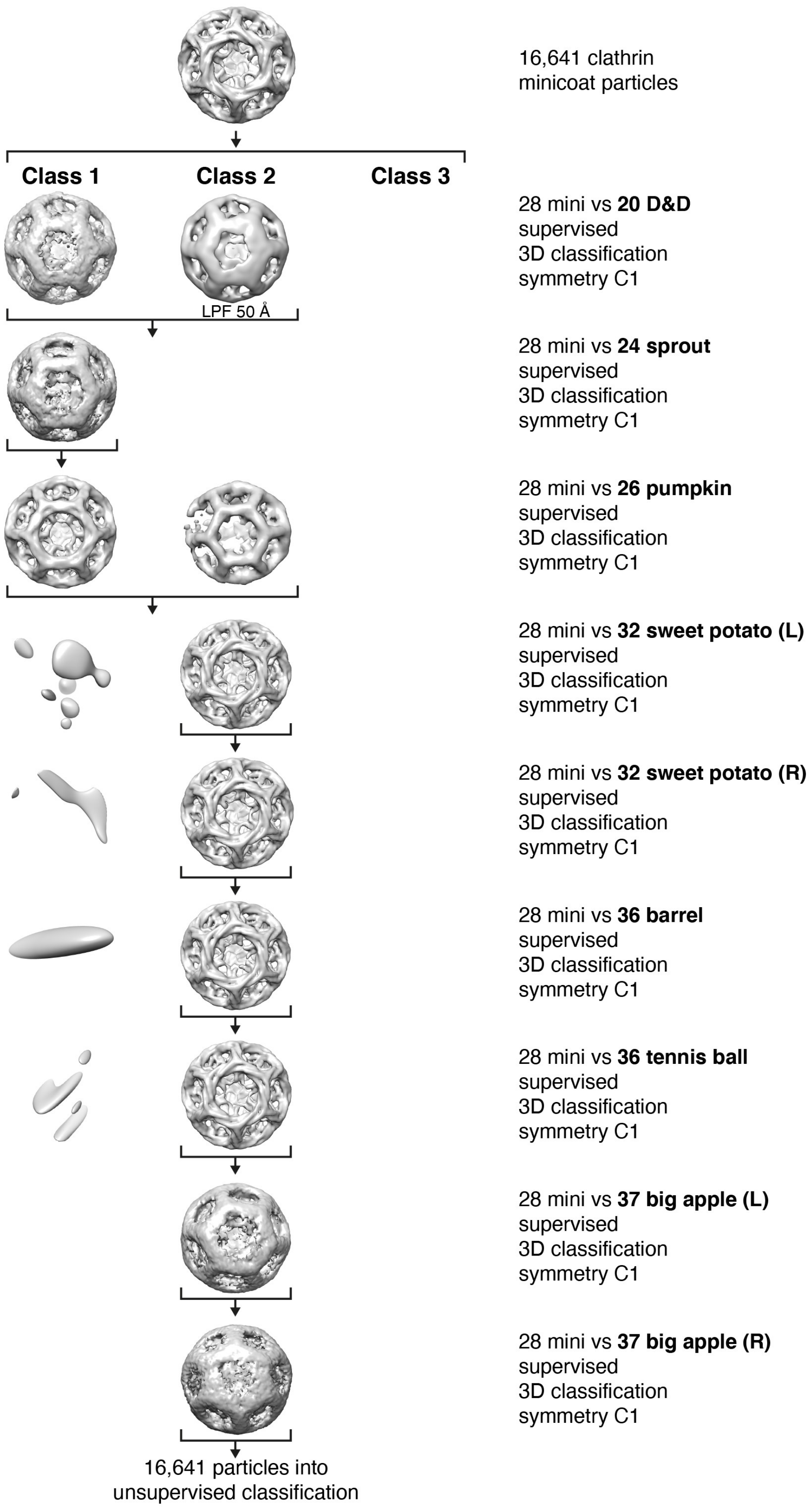
16,641 clathrin minicoat particles
Class 1
Class 2
Class 3
28 mini vs 20 D&D supervised 3D classification symmetry C1
LPF 50 Å
28 mini vs 24 sprout supervised 3D classification symmetry C1
28 mini vs 26 pumpkin supervised 3D classification symmetry C1
28 mini vs 32 sweet potato (L) supervised 3D classification symmetry C1
28 mini vs 32 sweet potato (R) supervised 3D classification symmetry C1
28 mini vs 36 barrel supervised 3D classification symmetry C1
28 mini vs 36 tennis ball supervised 3D classification symmetry C1
28 mini vs 37 big apple (L) supervised 3D classification symmetry C1
28 mini vs 37 big apple (R) supervised 3D classification symmetry C1
16,641 particles into unsupervised classification

Appendix Figure S1. Supervised, hierarchical 3D classification scheme in RELION.

To identify particles most stably associated with the minicoat cage type, the 16 641 clathrin cage particles which were classified as a minicoat cage type during a supervised, asymmetric 3D classification (see Supplementary Figure S1), were input into a hierarchical 3D classification scheme in RELION. A total of 9, separate, 3D classifications were conducted whereby particles were classified against 2 reference structures: a minicoat and one of the nine other geometric references in the cage library. Only the particles classifying as a minicoat cage type were retained and input into the next 3D classification (indicated by arrows). If the 3D output was of poor quality, the volume was low-pass filtered (LPF) to aid identification of cage architecture. The output of each classification can be seen in each row of the figure.

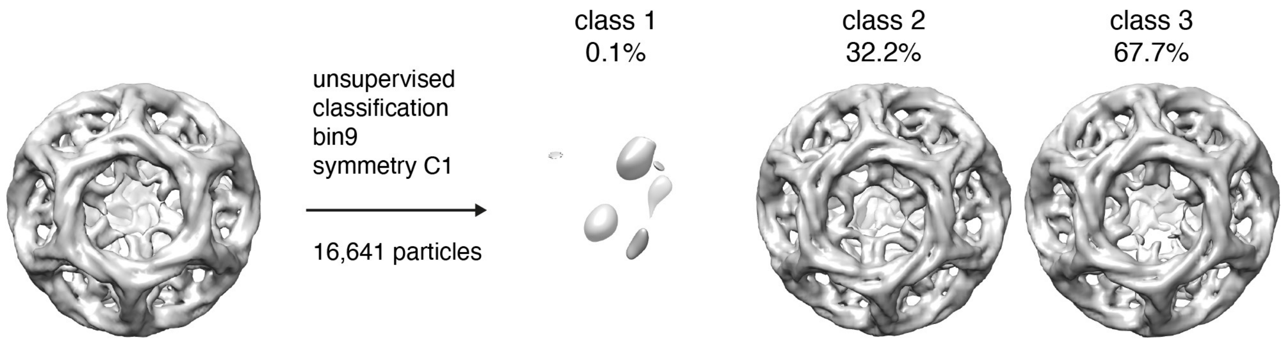

Appendix Figure S2. Unsupervised 3D classification in RELION.

Unsupervised classification of 16 641 minicoat particles. Output reconstructions were compared with the input supervised 3D classification volume. Aside from opposite handedness, classes 2 and 3 were architecturally identical to the input volume.

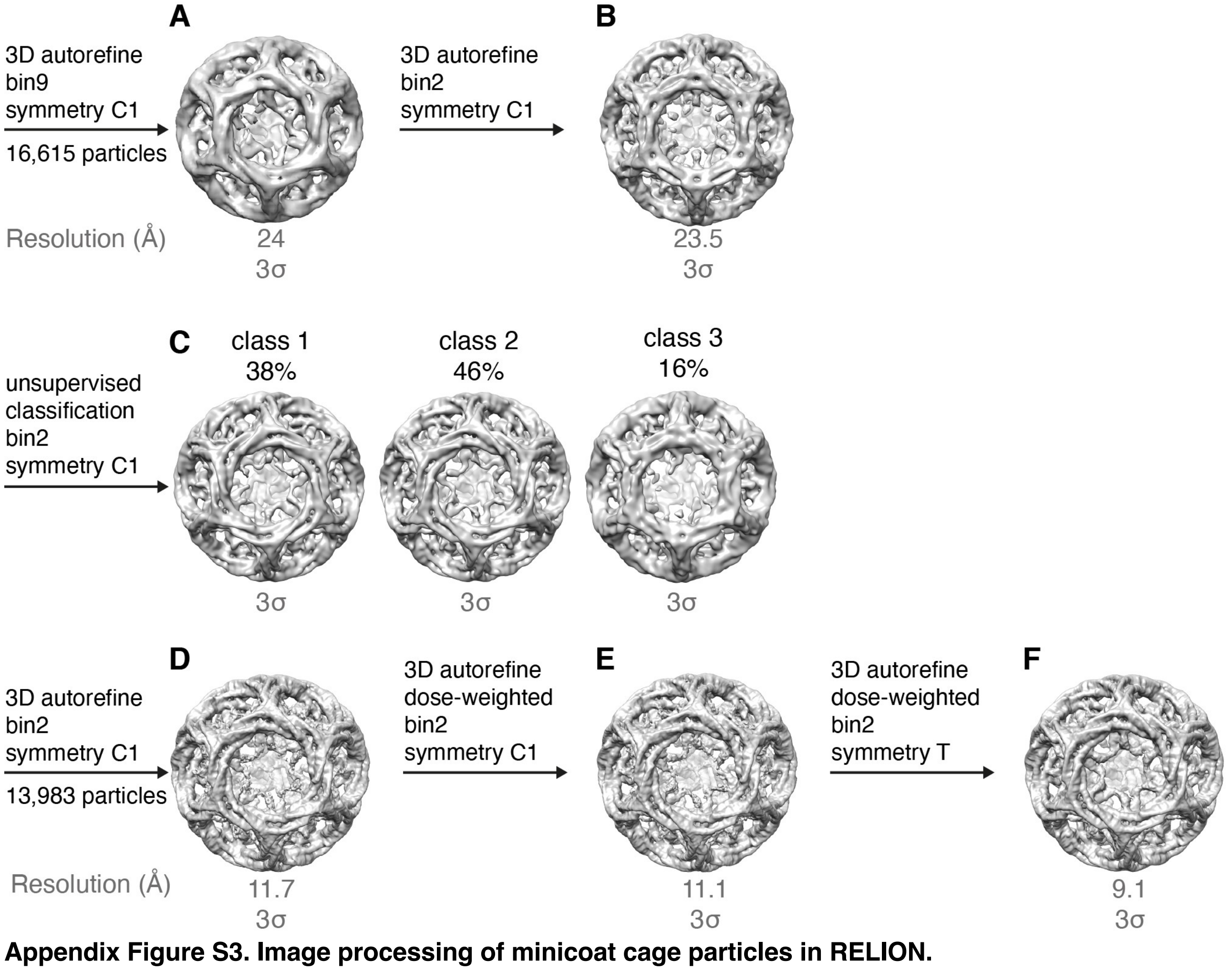

Appendix Figure S3. Image processing of minicoat cage particles in RELION.

(**A**) Initial 3D auto refinement of 16 615 minicoat particles yielded a 24 Å volume with minicoat architecture. (**B**) Unbinning of the particles from A to 2.78 Å px^{-1} and input into another 3D auto refinement produced a minicoat volume with mixed handedness. (**C**) To identify any minicoat particles of poor quality, particles from B were input into an unsupervised 3D classification. Class 3 contained particles that failed to produce a whole minicoat cage structure; therefore, only particles from classes 1 and 2 were taken to the next stage of image processing. D, E and F represent the final 3 rounds of 3D auto refinement with input parameters stated on the figure. A 9 Å minicoat cage structure was the final output.

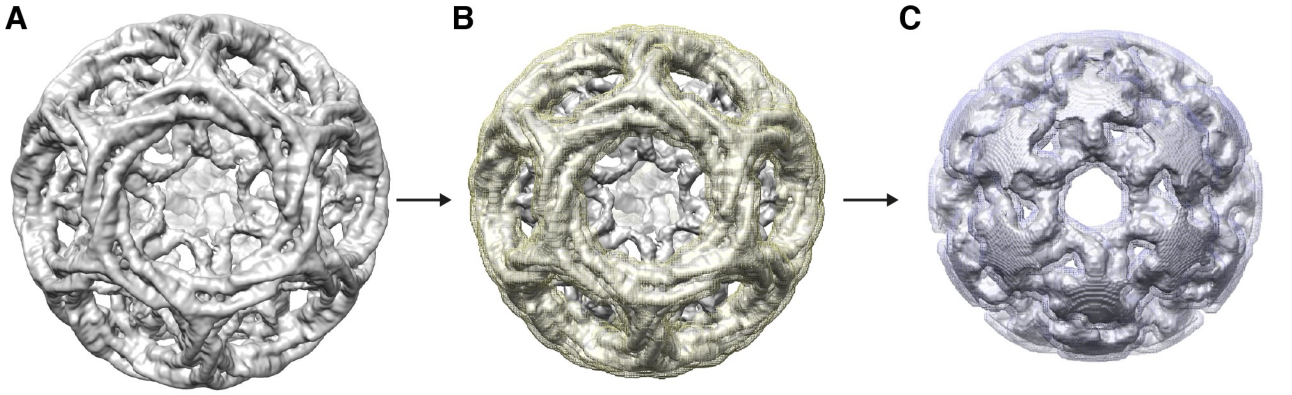

Appendix Figure S4. Signal subtraction of outer minicoat cage region.

(**A**) 9 Å minicoat cage structure at 3σ contour level. A soft mask (marked yellow in B) was applied to A to identify the protein density to be subtracted. The remaining density was input into a masked (marked purple in C), unsupervised 3D classification in RELION.
