# Supplementary material for: Multi‐modal adaptor‐clathrin contacts drive coated vesicle assembly
Source: EMBO J. 2021 Sep 6;40(19):e108795. doi: 10.15252/embj.2021108795 (PMC8488560; doi:10.15252/embj.2021108795)
Supplement: Supplementary file 2 — Expanded View Figures PDF [file EMBJ-40-e108795-s001.pdf]

Expanded View Figures

Figure EV1. Clathrin-β2HA reconstitution, data collection and processing.

A Clathrin triskelia (3 μM) were assembled in the presence of increasing concentrations (3–240 μM) of β2-adaptin$_{616\text{-}951}$ (β2HA). Clathrin assemblies were pelleted and analysed by SDS–PAGE to determine the amount of clathrin (CHC and CLCa/b) and β2HA in the pellet (P) and supernatant (S) fractions.

B Densitometry of gels in A shows that increasing amounts of β2HA pelleted with clathrin during the reconstitution experiments, with a 60-fold excess of adaptor protein yielding the maximum amount of clathrin-binding.

C Negative stain TEM analysis of clathrin cages reconstituted with a 60-fold excess (240 μM) of β2HA. Scale bar = 200 nm.

D A representative cryo-electron micrograph (left) at −1.4 μm defocus and the corresponding power spectrum indicating the information content at high spatial frequencies (right).

E 2D class averages of classes that were selected for 3D classification in RELION.

F Particle occupancy of the 10 classes obtained with supervised, asymmetric 3D classification in RELION.

G 3D surface representations of the 3 clathrin cages generated from the supervised, asymmetric 3D classification of clathrin cage particles. Their colour corresponds to the class occupancy data shown in panel F. Only the orange cage was reconstructed in full, enabling its cage geometry to be confirmed as minicoat.

© 2021 The Authors

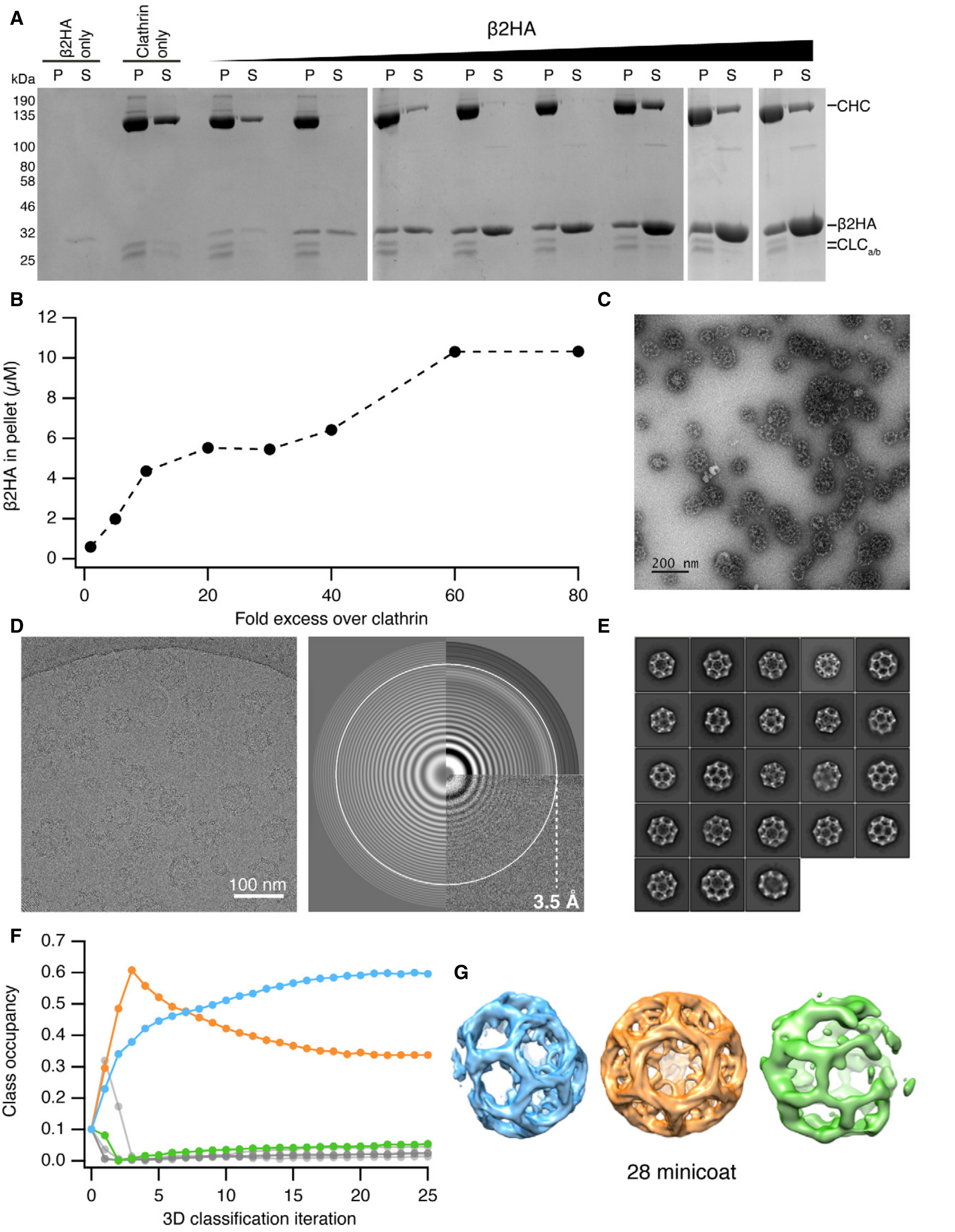

Figure EV1.

 © 2021 The Authors

Figure EV2. Unsupervised, masked 3D classification of signal-subtracted minicoat cage particles.

Output of unsupervised, masked 3D classification of signal-subtracted minicoat cage particles. Particles were separated into 20 classes: hexagonal faces (representative of average class quality for all remaining polygonal faces) are shown for each class with the corresponding refinement (at 3σ contour level) shown below.

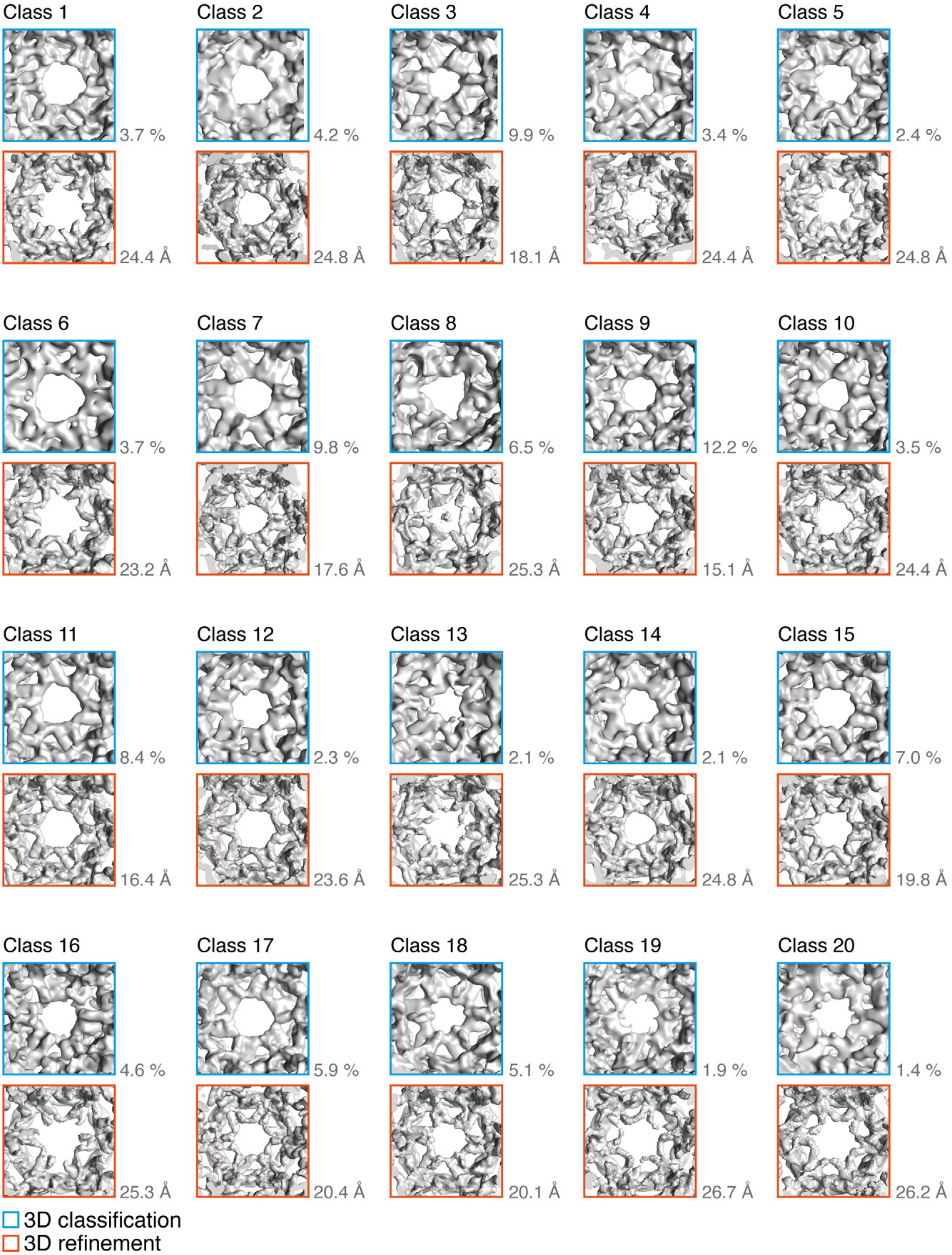

Figure EV2.

 © 2021 The Authors

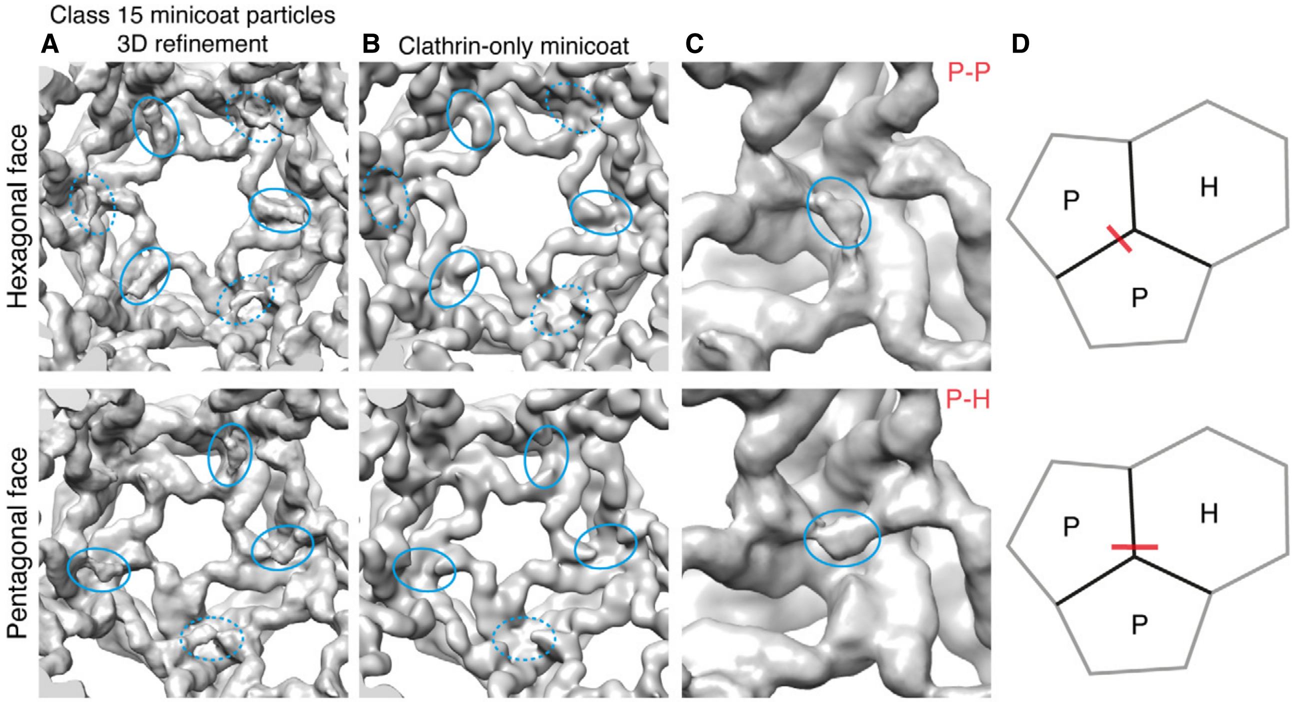

Figure EV3. Locating and sub-classifying β2HA density in class 15 of masked, 3D classification output.

A Representative hexagonal and pentagonal faces for class 15 3D auto refinement. Solid blue ellipses highlight new density seen following masked, 3D classification within a given polygonal face. Dashed ellipses highlight densities connecting adjacent polygonal faces.

B Representative hexagonal and pentagonal faces for clathrin-only minicoat cage (low pass filtered to 20 Å). Equivalent positions to those in column A are marked in ellipses, highlighting the lack of density in these regions.

C, D Density cross-linking terminal domains from two, adjacent pentagonal faces (denoted P-P) is marked in blue ellipse. Density cross-linking terminal domains from adjacent hexagonal and pentagonal faces (denoted P-H) is marked in blue ellipse. The geometric context of P-P and P-H densities is depicted in D.

Figure EV4. Localized reconstruction of minicoat cage particles.

A Asymmetric 3D auto refinement of hub regions for all 26,624 minicoat particles from class 15 yielded a 9.7 Å resolution volume. 180° rotation of this asymmetric unit revealed a single β2-appendage connecting two or three terminal domains (panel below and inset). Local resolution of this region ranged from 12 Å to 16 Å (G).

B, C Sub-classification of hub particles based on geometric context. P-P and H-P hubs (defined in Fig EV3) yielded volumes resolved at 10.5 Å and 10.1 Å volumes, respectively. The local resolution of the lower hub regions was approximately 16 Å (H and I). Both volumes gave improved definition of the β2-appendage density.

D–F FSC plots for maps shown in A, B and C, respectively. The resolution cut-off used was at a correlation value of 0.143.

G–I Shows maps A, B and C, respectively, coloured by local resolution. The scale gives the local resolution in Å.

© 2021 The Authors

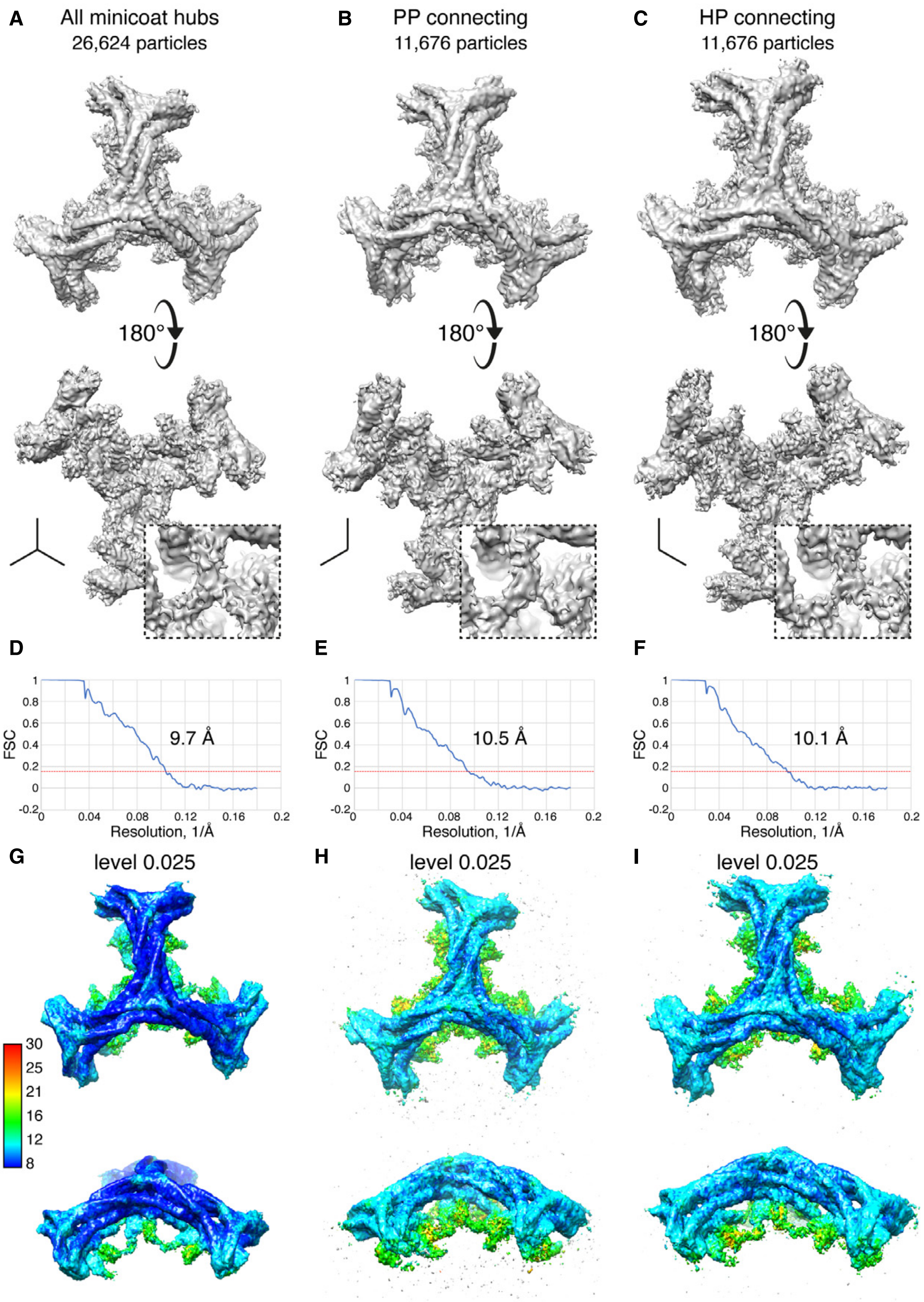

Figure EV4.

 © 2021 The Authors

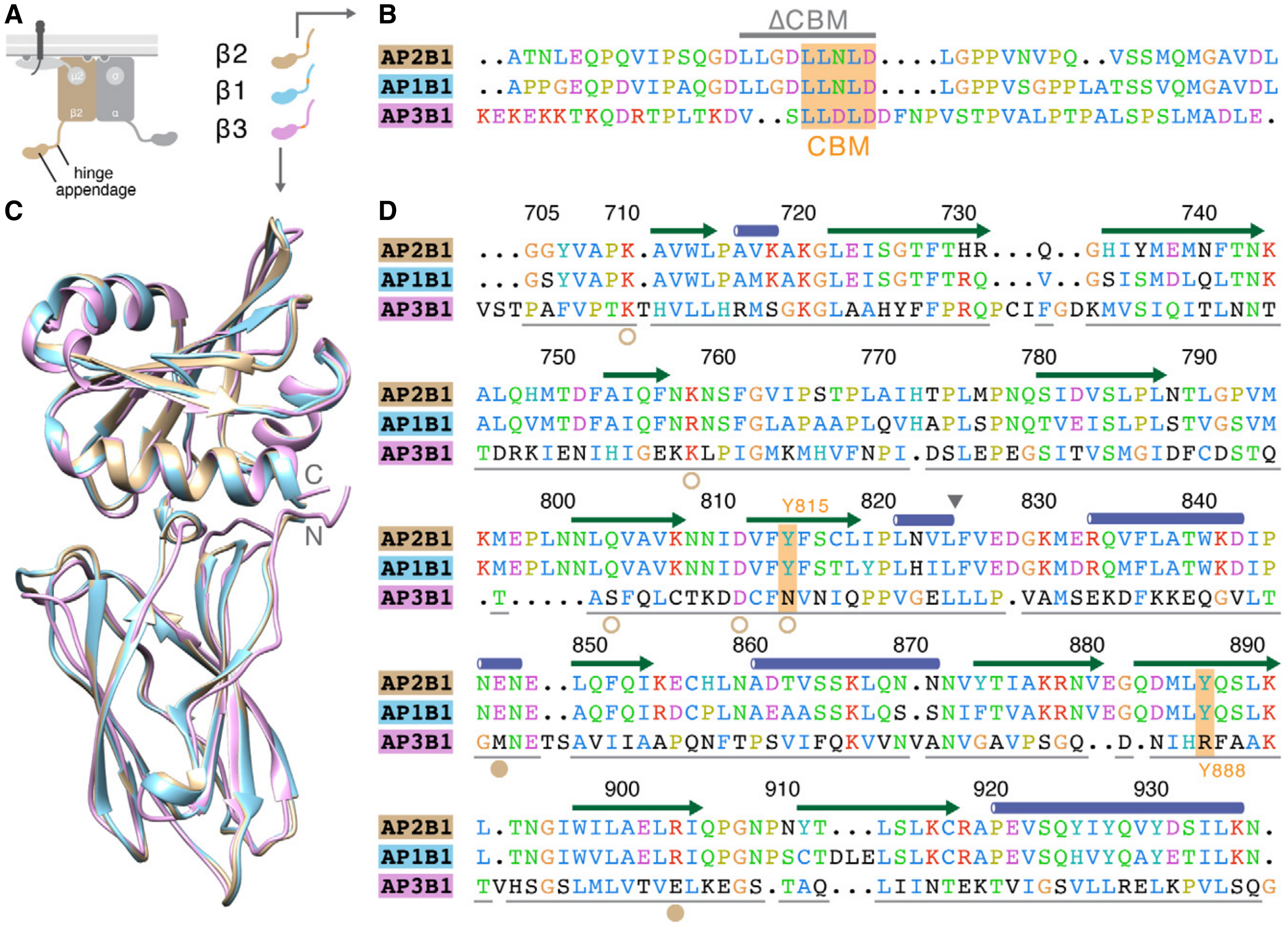

Figure EV5. Sequence comparison between β2 and β1 or β3.

A Schematic diagram of the AP2 complex showing the position of hinge and appendage of the β2 subunit. The colour coding for hinge and appendage of β2, β1 and β3 is shown (left) with the position of the clathrin-box motif (orange).

B Alignment of a section of the hinge region of β2, β1 and β3 containing the LL[D/N]LD clathrin-box motif (orange, CBM). The region deleted in ΔCBM construct is indicated by a grey line. Residues, β2 612-655, β1 613–658 and β3 835-883, are coloured according to property. Residues highlighted by BAlaS are indicated (platform residues, filled circles; sandwich residues, open circles).

C Overlay of β2, β1 and β3 appendage structures. The β2 structure is PDB code 2G30, β1 was created using MODELLER with 2G30 as a template and β3 was created using I-TASSER using 1E42 as a template.

D Alignment of the appendage regions shown in C. Structural features and numbering of β2 is shown above. Triangle indicates the separation between the two lobes of the appendage. The position of Tyr 815 and Tyr 888 is indicated in orange. Grey lines indicate structural alignment. Residues are shown coloured by property, and black residues are shown against consensus.
