# Supplementary material for: Multi‐modal adaptor‐clathrin contacts drive coated vesicle assembly
Source: EMBO J. 2021 Sep 6;40(19):e108795. doi: 10.15252/embj.2021108795 (PMC8488560; doi:10.15252/embj.2021108795)
Supplement: Supplementary file 3 [file EMBJ-40-e108795-s003.pdf]

THE EMBO JOURNAL

Multi-modal adaptor-clathrin contacts drive coated vesicle assembly

Sarah Smith, Gabrielle Larocque, Katherine Wood, Kyle Morris, Alan Roseman, Richard Sessions, Stephen Royle, and Corinne Smith
DOI: 10.15252/embj.2021108795

Corresponding author(s): Stephen Royle (S.J.Royle@warwick.ac.uk) , Corinne Smith (corinne.smith@warwick.ac.uk), Stephen Royle (S.J.Royle@warwick.ac.uk)

Review Timeline:

Submission Date:	23rd May 21
Editorial Decision:	29th Jun 21
Revision Received:	16th Jul 21
Editorial Decision:	28th Jul 21
Revision Received:	30th Jul 21
Accepted:	3rd Aug 21

Editor: Elisabetta Argenzio

Transaction Report:

(Note: With the exception of the correction of typographical or spelling errors that could be a source of ambiguity, letters and reports are not edited. Depending on transfer agreements, referee reports obtained elsewhere may or may not be included in this compilation. Referee reports are anonymous unless the Referee chooses to sign their reports.)

1st Editorial Decision 29th Jun 2021

Thank you for submitting your study entitled "Multi-modal adaptor-clathrin contacts drive coated vesicle assembly" [EMBOJ-2021-108795]. The manuscript has been assessed by two reviewers, whose reports are enclosed below.

As you will see, the referees find your study potentially interesting. However, they also raise some points that need to be addressed before they can support publication here.

Given the interest of your findings, I am pleased to invite submission of a manuscript revised as indicated in the reports attached herein. I should also add that it is our policy to allow only a single round of major revision. Therefore, acceptance of your manuscript will depend on the completeness of your responses in this revised version.

We generally grant three months as standard revision time. As we are aware that many laboratories cannot function at full capacity owing to the COVID-19 pandemic, we may relax this deadline. Also, we have decided to apply our 'scooping protection policy' to the time span required for you to fully revise your manuscript and address the experimental issues highlighted herein. Nevertheless, please inform us as soon as a paper with related content is published elsewhere.

Referee #1:

In Smith et al., the authors describe new results on the significance of AP2 in binding and organizing the assembly of clathrin. Specifically, they focus on the role of β2 hinge and appendage domains. They show that the β2 hinge and appendage domains are critical for CME using the hotwired endocytosis technique that they previously developed and showed that the β2 hinge could be replaced with the β3 hinge, but the β2 appendage was indispensable. They then characterized the binding of the β2 appendage using cryo-EM and showed that it binds multiple different sites on the clathrin cage. Altogether, the results and analyses are of high quality. A significant result is that they rationalize a discrepancy between two recently published clathrin+adaptor structures, Paraan et al. and Kovtun et al. One weakness is that their cryo-EM results are relatively low resolution, but on the other hand high resolution is not required for the conclusions they are making. A few weaknesses were identified as well as some suggestions, and these are listed in no particular order below.

1. Figure 1 D i and ii don't add much and are confusing. Perhaps instead use a 2D illustration?
2. In the introduction- in the first paragraph - when talking about sandwich domain and platform domain and the residues- they could be pointed out in the corresponding figures.
3. A little more description of how the hot-wired endocytosis technique works would make the manuscript read better
4. Figure 2A, consider dropping the top panel and instead having a schematic where you have the β2 and β3 appendages colored as they are in Fig. 2B. Also maybe consider illustrating a summary of the results in the Fig. 2A illustration
5. The lower resolution compared to recent clathrin structures is surprising. Do the authors think the β2HA is somehow introducing more heterogeneity or something like that?
6. Under 'Finding b2HA in clathrin-b2HA minicoats': 'Fig 4D' has been mentioned but there is no Fig 4D image
7. For Figure 6C, it would be nice to put a note like you did on Figure 4 indicating whether it's an inside out or outside in view.
8. The hotwiring results are very clear, and the combination of the computational binding analysis and testing the findings with mutagenesis with the hotwiring assay is seen as a strength.
9. In supplementary fig S9, residue numbers that have been mentioned can be depicted in the figure and based on what 'property' they have been color coded.
10. Buffer composition can be mentioned along with the purification procedure. Buffers like 'HKM buffer' and 'elution buffer' were mentioned under buffer composition but not mentioned where they were used.
11. For supplementary fig 1A, the meaning of the terms 'S' and 'P' as supernatant and pellet should be mentioned in the legend.

Referee #2:

In this paper by Smith, Royle, and Smith the interactions between the AP2 B2 subunit and the clathrin lattice is examined with cryoEM and synthetic cell biology. To summarize, the authors identify the B2-appendange domain as a key driver of clathrin assembly on the plasma membrane. With purified single basket cryoEM structures, the authors propose that the AP2 appendage has multi-modal binding interactions and can act to cross link or bridge the N-terminal domains of adjacent triskelions to promote lattice assembly. The Y815 residue in the sandwich domain along with the clathrin box is further shown to be primary to drivers of the interaction. In general, I find this paper well done, carefully analyzed, and interesting. It is a nice combination of structural biology and

cell biology to answer a current gap in the field of membrane traffic. The data generated in this work will lead to additional studies and tools useful to disentangle the complex assembly of the clathrin lattice at the plasma membrane. The paper does have limitations, but most of my criticisms should be able to addressed in a straightforward manner. My specific comments follow.

1. "was not able to induce endocytosis" pg 2. Here, I would argue that the authors have only shown that the appendage domain can promote assembly of the lattice and not induce endocytosis. These sites are not shown to be functional endocytic structures that are internalized. They are only shown to assemble as diffraction-limited spots on the plasma membrane. Please adjust the text to clarify this idea.

2. Are the clathrin sites on the membrane that are formed in the "hot-wire" system curved or flat? Are they all the same size? Are they organized lattices? Some EM or super-resolution (platinum replica or thin section or 2D/3D-storm) could be nice to show how the lattice on the membrane relates to the cryoEM structures done on the purified mini-coats. The mini-baskets solved by cryoEM could be very different than the ones formed on the membrane in cells. This is not absolutely necessary but would be nice and make for a more cohesive paper.

3. The density docking in Figure 4C appears to position 1E42 with Y815 facing away from 1BPO. It is difficult to see in this presentation, but if that is case, why is this docking different than the docking shown in the later figures?

4. The potential salt-bridges in the model (Figure 6) are intriguing. Can the authors further comment on the idea that these are important interactions in the complex? Again, it is difficult to see without the pdb model. Could the authors please expand on the discussion and figures to aid future mutagenesis (double-mutant cycle analysis for example) to explore this interface?

5. Can you please highlight where aa815 and aa888 are in the docked structure in Figure 6? As these are the key residues it would be helpful to highlight them.

6. A major concern that should be addressed in the text is how the structure of the AP2 fragment relates to structures of intact and complete AP2/Clathrin complexes. Is it possible that the unconstrained nature of the fragment in both the cryoEM and hotwire system allows for interactions that are not physiological? Please comment on this caveat in the text.

We thank Referee #1 for their time and constructive criticism of our paper.

Referee #1:

In Smith et al., the authors describe new results on the significance of AP2 in binding and organizing the assembly of clathrin. Specifically, they focus on the role of β2 hinge and appendage domains. They show that the β2 hinge and appendage domains are critical for CME using the hotwired endocytosis technique that they previously developed and showed that the β2 hinge could be replaced with the β3 hinge, but the β2 appendage was indispensable. They then characterized the binding of the β2 appendage using cryo-EM and showed that it binds multiple different sites on the clathrin cage. Altogether, the results and analyses are of high quality. A significant result is that they rationalize a discrepancy between two recently published clathrin+adaptor structures, Paraan et al. and Kovtun et al. One weakness is that their cryo-EM results are relatively low resolution, but on the other hand high resolution is not required for the conclusions they are making. A few weaknesses were identified as well as some suggestions, and these are listed in no particular order below.
1. Figure 1 D i and ii don't add much and are confusing. Perhaps instead use a 2D illustration?

We think Figure 1D is important because it shows the positioning of terminal domains in the context of the clathrin cage and this information is needed to understand the later figures in the paper. However, we appreciate that cage geometry is confusing to non-specialist readers. So in response to this comment, we have introduced a schematic version of the inner views presented in Figure 1D ii which help to orient the reader to clathrin interactions that are pertinent to the study.

2. In the introduction- in the first paragraph - when talking about sandwich domain and platform domain and the residues- they could be pointed out in the corresponding figures.

Thank you, done.

3. A little more description of how the hot-wired endocytosis technique works would make the manuscript read better

As described below, we have altered the schematic figure in Figure 2A and its legend to describe the method in more detail. We have also added a sentence to where the technique is introduced in order to describe the method better:
These puncta move inside the cell, away from the plasma membrane and we have shown previously that they are clathrin-coated vesicles that have pinched off from the surface and are competent for traffic inside the cell (Wood et al., 2017).

4. Figure 2A, consider dropping the top panel and instead having a schematic where you have the β2 and β3 appendages colored as they are in Fig. 2B. Also maybe consider illustrating a summary of the results in the Fig. 2A illustration

The Referee was correct that the top part of Figure 2A was unnecessary. The revised figure shows AP2 and AP3 in order to introduce the color scheme used in part B. In this schematic diagram, we have added the fact that the plasma membrane “anchor” used for hot-wiring is labelled extracellularly with a fluorescent antibody. This allows us to detect endocytosis (which was not clear to Referee #2). We have not added a summary of results since 2A is before the data and results are presented (2B and 2C).

5. The lower resolution compared to recent clathrin structures is surprising. Do the authors think the β2HA is somehow introducing more heterogeneity or something like that?

Yes, there is much more heterogeneity in the clathrin-β2HA sample compared with other samples and we agree with the referee that this must affect resolution. We have included some comments on this at the beginning of the results section entitled ‘Finding β2HA in clathrin-β2HA minicoats’:
Association of β2HA with clathrin cages may increase sample heterogeneity either through effects on the cage structure itself or through variations in mode of binding, ultimately affecting resolution. In addition, clathrin terminal domain flexibility may result in weaker density in the terminal domain and linker region (Fotin et al., 2004; Morris et al., 2019).

6. Under 'Finding b2HA in clathrin-b2HA minicoats': 'Fig 4D' has been mentioned but there is no Fig 4D image

Thank you for spotting this. It has been corrected to Figure 4C.

7. For Figure 6C, it would be nice to put a note like you did on Figure 4 indicating whether it's an inside out or outside in view.

We have added labels like this to Figure 6C and to Figures 1 and 5.

8. The hotwiring results are very clear, and the combination of the computational binding analysis and testing the findings with mutagenesis with the hotwiring assay is seen as a strength.

We were really pleased with this comment!

9. In supplementary fig S9, residue numbers that have been mentioned can be depicted in the figure and based on what 'property' they have been color coded.

Thank you for this suggestion. We have added circles to denote those residues identified by BAlaS. This figure is now elevated to EV5 in the paper so that readers can visualize the conservation and properties of these residues closer to Figure 6.

10. Buffer composition can be mentioned along with the purification procedure. Buffers like 'HKM buffer' and 'elution buffer' were mentioned under buffer composition but not mentioned where they were used.

We have edited the ‘Protein expression and purification’ section of the Materials and Methods so that all buffer compositions are now referred to.

11. For supplementary fig 1A, the meaning of the terms 'S' and 'P' as supernatant and pellet should be mentioned in the legend.

Thank you. This is done (Figure is now EV1).

We thank Referee #2 for their time and comment on our manuscript. We were pleased that, like Referee #1, they were complimentary about our work.

Referee #2:

In this paper by Smith, Royle, and Smith the interactions between the AP2 B2 subunit and the clathrin lattice is examined with cryoEM and synthetic cell biology. To summarize, the authors identify the B2-appendange domain as a key driver of clathrin assembly on the plasma membrane. With purified single basket cryoEM structures, the authors propose that the AP2 appendage has multi-modal binding interactions and can act to cross link or bridge the N-terminal domains of adjacent triskelions to promote lattice assembly. The Y815 residue in the sandwich domain along with the clathrin box is further shown to be primary to drivers of the interaction. In general, I find this paper well done, carefully analyzed, and interesting. It is a nice combination of structural biology and cell biology to answer a current gap in the field of membrane traffic. The data generated in this work will lead to additional studies and tools useful to disentangle the complex assembly of the clathrin lattice at the plasma membrane. The paper does have limitations, but most of my criticisms should be able to addressed in a straightforward manner. My specific comments follow.

1. "was not able to induce endocytosis" pg 2. Here, I would argue that the authors have only shown that the appendage domain can promote assembly of the lattice and not induce endocytosis. These sites are not shown to be functional endocytic structures that are internalized. They are only shown to assemble as diffraction-limited spots on the plasma membrane. Please adjust the text to clarify this idea.

We are sorry that we did not describe our method in enough detail (a point also raised by Referee #1). Our method certainly *does* induce endocytosis. In our previous paper (Wood et al., J Cell Biol, 2017), we showed that the puncta we analyze here are endocytic clathrin-coated vesicles. Briefly:

1. the puncta that are formed during hot-wiring contain a fluorescent antibody which was applied extracellularly and this can be distinguished from cargo (and pits) still at the surface.
2. we showed by EM that the puncta that form are clathrin coated vesicles pinched off from the surface that contain the extracellularly applied antibody.
3. the hot-wired endocytic vesicles that form are motile.
4. the hot-wired endocytic vesicles are trafficked intracellularly, colocalizing with Rabs on the recycling pathway.

In the current manuscript we show that hot-wired endocytic structures are internalized because they contain fluorescent antibody that was applied extracellularly. Regarding the Referee's alternative idea – that the puncta are still attached to the surface – we direct them to Figure 2B where a) the yellow spots are deep in the cell, well away from the plasma membrane and b) the amount of surface label has been reduced for FKGP-β2HA-GFP and FKBP-β3Hβ2A-GFP because it has been removed from the surface by hot-wiring.

Having said all this, our description was clearly not sufficient to explain our method. Therefore, we have enhanced the schematic diagram in Figure 2A and its legend to

show that we are indeed inducing the uptake of surface-labelled protein into vesicles. We have also edited the description of the method where it was first introduced: These puncta move inside the cell, away from the plasma membrane and we have shown previously that they are clathrin-coated vesicles that have pinched off from the surface and are competent for traffic inside the cell (Wood et al., 2017).

2. Are the clathrin sites on the membrane that are formed in the "hot-wire" system curved or flat? Are they all the same size? Are they organized lattices? Some EM or super-resolution (platinum replica or thin section or 2D/3D-storm) could be nice to show how the lattice on the membrane relates to the cryoEM structures done on the purified mini-coats. The mini-baskets solved by cryoEM could be very different than the ones formed on the membrane in cells. This is not absolutely necessary but would be nice and make for a more cohesive paper.

As described above, our previous work confirmed that the hot-wired system produces clathrin-coated vesicles and not stalled lattices on membranes. In Wood et al., 2017 we showed that hot-wired clathrin-coated vesicles are morphologically similar to regular clathrin-coated vesicles by electron microscopy. We think it is reasonable to assume that our cryoEM structures are similar to CCVs found in cells whether hot-wired or regular due to the similarities that exist between structures of *in vitro* clathrin assemblies (our study; Morris et al., Nat Struct Mol Biol, 2019) and those of purified clathrin-coated vesicles (Cheng et al., J Mol Biol, 2007; Heymann et al., J Struc Biol, 2013; Paraan et al., Sci Adv 2020). A comparison of *in situ* coated vesicles by cryo-electron tomography with *in vitro* clathrin assemblies would be interesting, however achieving the resolution necessary for a meaningful comparison would be a substantial undertaking and is not feasible in the present study.

3. The density docking in Figure 4C appears to position 1E42 with Y815 facing away from 1BPO. It is difficult to see in this presentation, but if that is case, why is this docking different than the docking shown in the later figures?

Thank you for highlighting this. In fact, the low resolution of the density shown in Figure 4C (cyan-colored volume) precludes unambiguous, automated fitting of the β2 appendage in the surplus density either side of the clathrin terminal domain. This means we are unable to determine the *exact* orientation of the β2 appendages in this particular class of particles. We did not intend to give this impression, so to clarify, we have replaced the atomic models of the β2 appendages with low resolution, meshed surface volumes, which shows that the density available can accommodate protein of the size and shape of the β2 appendage but does not suggest we are proposing its precise orientation within the density.

4. The potential salt-bridges in the model (Figure 6) are intriguing. Can the authors further comment on the idea that these are important interactions in the complex? Again, it is difficult to see without the pdb model. Could the authors please expand on the discussion and figures to aid future mutagenesis (double-mutant cycle analysis for example) to explore this interface?

This is an excellent point. We have added further comment on this in the Results section (at the end of the section entitled 'Defining β2-appendage interactions with clathrin terminal domains')
Interestingly in our model Lys 140, Lys 189 and Asp 187 form salt bridge contacts with Glu 847, Glu 849 and Arg 904 respectively. In the α-appendage control only Glu 849 and Lys 189 form a plausible salt bridge, suggesting a role for Lys140/Glu 847 and Asp187/Arg 904 salt bridges in the specificity of this interaction.

and to the discussion:
Our *in silico* analysis indicates that, in one mode, the platform site of β2HA does make contact with the terminal domain, and that two pairs of salt bridges (Lys140/Glu847 and Asp187/Arg904) may stabilize this interaction. We interpret our hot-wiring results to mean that this contact occurs only after binding of the hinge and the sandwich site. Further studies testing the effect of mutagenesis at this interface would help determine whether clathrin binding at this site is of functional importance.

5. Can you please highlight where aa815 and aa888 are in the docked structure in Figure 6? As these are the key residues it would be helpful to highlight them.

Figure 6, panel C, has been amended so that amino acid residues Tyr888 and Tyr815 are now shown and highlighted in dashed ellipses.

6. A major concern that should be addressed in the text is how the structure of the AP2 fragment relates to structures of intact and complete AP2/Clathrin complexes. Is it possible that the unconstrained nature of the fragment in both the cryoEM and hotwire system allows for interactions that are not physiological? Please comment on this caveat in the text.

As requested we have added a comment on this in the discussion:
In comparing our results with those of Kovtun *et al* and Paraan *et al* we see that differing sample conditions such as inclusion of membrane and cargo (Kovtun et al., 2020) or use of coated vesicles purified from source (Paraan et al., 2020) do not explain differences in β-appendage binding location since in our study with only two purified protein components we see multiple binding locations that either confirm (Kovtun et al., 2020) or are similar to (Paraan et al., 2020) those seen in more complex systems. One interpretation of this is that the long linker domain on β2-adaptin permits the β-appendage to move freely, even within a coated vesicle, and form stable interactions unencumbered by the need for additional components or a specific orientation. Further structural and functional studies in systems that more closely reflect the physiological environment will be needed to confirm this.

1st Revision - Editorial Decision 28th Jul 2021

Thank you for submitting your revised study.

We find that the referees' criticisms have been adequately addressed. However, there are few editorial issues concerning the text and the figures that I need you to address before we can officially accept your manuscript.

2nd Revision - Editorial Decision 3rd Aug 2021

I am pleased to inform you that your manuscript has been accepted for publication in The EMBO Journal.

EMBO PRESS

YOU MUST COMPLETE ALL CELLS WITH A PINK BACKGROUND ↓

PLEASE NOTE THAT THIS CHECKLIST WILL BE PUBLISHED ALONGSIDE YOUR PAPER

Corresponding Author Name: Corinne Smith and Stephen Royle
Journal Submitted to: EMBO J
Manuscript Number: EMBOJ-2021-108795

USEFUL LINKS FOR COMPLETING THIS FORM

http://www.antibodypedia.com
http://1degreebio.org
http://www.equator-network.org/reporting-guidelines/improving-bioscience-research-repor
http://grants.nih.gov/grants/olaw/olaw.htm
http://www.mrc.ac.uk/Ourresearch/Ethicsresearchguidance/Useofanimals/index.htm
http://ClinicalTrials.gov
http://www.consort-statement.org
http://www.consort-statement.org/checklists/view/32-consort/66-title
http://www.equator-network.org/reporting-guidelines/reporting-recommendations-for-tumo
http://datadryad.org
http://figshare.com
http://www.ncbi.nlm.nih.gov/gap
http://www.ebi.ac.uk/ega
http://biomodels.net/
http://biomodels.net/miriam/
http://jjj.biochem.sun.ac.za
https://osp.od.nih.gov/biosafety-biosecurity-and-emerging-biotechnology/
http://www.selectagents.gov/

Reporting Checklist For Life Sciences Articles (Rev. June 2017)

This checklist is used to ensure good reporting standards and to improve the reproducibility of published results. These guidelines are consistent with the Principles and Guidelines for Reporting Preclinical Research issued by the NIH in 2014. Please follow the journal's authorship guidelines in preparing your manuscript.

A- Figures
1. Data
The data shown in figures should satisfy the following conditions:

- the data were obtained and processed according to the field's best practice and are presented to reflect the results of the experiments in an accurate and unbiased manner.
- figure panels include only data points, measurements or observations that can be compared to each other in a scientifically meaningful way.
- graphs include clearly labeled error bars for independent experiments and sample sizes. Unless justified, error bars should not be shown for technical replicates.
- if n< 5, the individual data points from each experiment should be plotted and any statistical test employed should be justified
- Source Data should be included to report the data underlying graphs. Please follow the guidelines set out in the author ship guidelines on Data Presentation.

2. Captions

Each figure caption should contain the following information, for each panel where they are relevant:

- a specification of the experimental system investigated (eg cell line, species name).
- the assay(s) and method(s) used to carry out the reported observations and measurements
- an explicit mention of the biological and chemical entity(ies) that are being measured.
- an explicit mention of the biological and chemical entity(ies) that are altered/varied/perturbed in a controlled manner.

- the exact sample size (n) for each experimental group/condition, given as a number, not a range;
- a description of the sample collection allowing the reader to understand whether the samples represent technical or biological replicates (including how many animals, litters, cultures, etc.).
- a statement of how many times the experiment shown was independently replicated in the laboratory.
- definitions of statistical methods and measures:
  - common tests, such as t-test (please specify whether paired vs. unpaired), simple χ2 tests, Wilcoxon and Mann-Whitney tests, can be unambiguously identified by name only, but more complex techniques should be described in the methods section;
  - are tests one-sided or two-sided?
  - are there adjustments for multiple comparisons?
  - exact statistical test results, e.g., P values = x but not P values < x;
  - definition of 'center values' as median or average;
  - definition of error bars as s.d. or s.e.m.

Any descriptions too long for the figure legend should be included in the methods section and/or with the source data.

In the pink boxes below, please ensure that the answers to the following questions are reported in the manuscript itself. Every question should be answered. If the question is not relevant to your research, please write NA (non applicable). We encourage you to include a specific subsection in the methods section for statistics, reagents, animal models and human subjects.

B- Statistics and general methods

Please fill out these boxes ↓ (Do not worry if you cannot see all your text once you press return)

1.a. How was the sample size chosen to ensure adequate power to detect a pre-specified effect size?	For cell biology experiments, previous work allowed us to determine sample size. For cryoEM a complete dataset is presented
1.b. For animal studies, include a statement about sample size estimate even if no statistical methods were used.	NA
2. Describe inclusion/exclusion criteria if samples or animals were excluded from the analysis. Were the criteria pre-established?	No inclusion/exclusion criteria were used
3. Were any steps taken to minimize the effects of subjective bias when allocating animals/samples to treatment (e.g. randomization procedure)? If yes, please describe.	NA
For animal studies, include a statement about randomization even if no randomization was used.	NA
4.a. Were any steps taken to minimize the effects of subjective bias during group allocation or/and when assessing results (e.g. blinding of the investigator)? If yes please describe.	NA
4.b. For animal studies, include a statement about blinding even if no blinding was done	NA
5. For every figure, are statistical tests justified as appropriate?	Yes
Do the data meet the assumptions of the tests (e.g., normal distribution)? Describe any methods used to assess it.	Yes
Is there an estimate of variation within each group of data?	For cell biology experimets. All data points are plotted and summary statistics are shown. One-way ANOVA was used to analyse the variance between each group.

Is the variance similar between the groups that are being statistically compared?	NA

C- Reagents

6. To show that antibodies were profiled for use in the system under study (assay and species), provide a citation, catalog number and/or clone number, supplementary information or reference to an antibody validation profile. e.g., Antibodypedia (see link list at top right), 1DegreeBio (see link list at top right).	
7. Identify the source of cell lines and report if they were recently authenticated (e.g., by STR profiling) and tested for mycoplasma contamination.	

* for all hyperlinks, please see the table at the top right of the document

D- Animal Models

8. Report species, strain, gender, age of animals and genetic modification status where applicable. Please detail housing and husbandry conditions and the source of animals.	NA
9. For experiments involving live vertebrates, include a statement of compliance with ethical regulations and identify the committee(s) approving the experiments.	NA
10. We recommend consulting the ARRIVE guidelines (see link list at top right) (PLoS Biol. 8(6), e1000412, 2010) to ensure that other relevant aspects of animal studies are adequately reported. See author guidelines, under 'Reporting Guidelines'. See also: NIH (see link list at top right) and MRC (see link list at top right) recommendations. Please confirm compliance.	NA

E- Human Subjects

11. Identify the committee(s) approving the study protocol.	NA
12. Include a statement confirming that informed consent was obtained from all subjects and that the experiments conformed to the principles set out in the WMA Declaration of Helsinki and the Department of Health and Human Services Belmont Report.	NA
13. For publication of patient photos, include a statement confirming that consent to publish was obtained.	NA
14. Report any restrictions on the availability (and/or on the use) of human data or samples.	NA
15. Report the clinical trial registration number (at ClinicalTrials.gov or equivalent), where applicable.	NA
16. For phase II and III randomized controlled trials, please refer to the CONSORT flow diagram (see link list at top right) and submit the CONSORT checklist (see link list at top right) with your submission. See author guidelines, under 'Reporting Guidelines'. Please confirm you have submitted this list.	NA
17. For tumor marker prognostic studies, we recommend that you follow the REMARK reporting guidelines (see link list at top right). See author guidelines, under 'Reporting Guidelines'. Please confirm you have followed these guidelines.	NA

F- Data Accessibility

18: Provide a "Data Availability" section at the end of the Materials & Methods, listing the accession codes for data generated in this study and deposited in a public database (e.g. RNA-Seq data: Gene Expression Omnibus GSE39462, Proteomics data: PRIDE PXD000208 etc.) Please refer to our author guidelines for 'Data Deposition'.<br><br>Data deposition in a public repository is mandatory for:<br>a. Protein, DNA and RNA sequences<br>b. Macromolecular structures<br>c. Crystallographic data for small molecules<br>d. Functional genomics data<br>e. Proteomics and molecular interactions	A Data Availability section is provided at the end of the Materials & Methods. It provides the accession codes for the maps and models in the Electron Microscopy Data Bank and the Protein Data Bank.
19. Deposition is strongly recommended for any datasets that are central and integral to the study; please consider the journal's data policy. If no structured public repository exists for a given data type, we encourage the provision of datasets in the manuscript as a Supplementary Document (see author guidelines under 'Expanded View' or in unstructured repositories such as Dryad (see link list at top right) or Figshare (see link list at top right).	NA
20. Access to human clinical and genomic datasets should be provided with as few restrictions as possible while respecting ethical obligations to the patients and relevant medical and legal issues. If practically possible and compatible with the individual consent agreement used in the study, such data should be deposited in one of the major public access-controlled repositories such as dbGAP (see link list at top right) or EGA (see link list at top right).	NA
21. Computational models that are central and integral to a study should be shared without restrictions and provided in a machine-readable form. The relevant accession numbers or links should be provided. When possible, standardized format (SBML, CellML) should be used instead of scripts (e.g. MATLAB). Authors are strongly encouraged to follow the MIRIAM guidelines (see link list at top right) and deposit their model in a public database such as Biomodels (see link list at top right) or JWS Online (see link list at top right). If computer source code is provided with the paper, it should be deposited in a public repository or included in supplementary information.	NA

G- Dual use research of concern

22. Could your study fall under dual use research restrictions? Please check biosecurity documents (see link list at top right) and list of select agents and toxins (APHIS/CDC) (see link list at top right). According to our biosecurity guidelines, provide a statement only if it could.	NA
